# Clinical Ocular Exposure Extrapolation for Ophthalmic Solutions Using PBPK Modeling and Simulation

**DOI:** 10.1007/s11095-022-03390-z

**Published:** 2022-09-23

**Authors:** Maxime Le Merdy, Farah AlQaraghuli, Ming-Liang Tan, Ross Walenga, Andrew Babiskin, Liang Zhao, Viera Lukacova

**Affiliations:** 1 Simulations Plus, Inc., 42505 10th Street West, Lancaster, CA 93534 USA; 2 Division of Quantitative Methods and Modeling, Office of Research and Standards, Office of Generic Drugs, Center for Drug Evaluation and Research, U.S. Food and Drug Administration, 10903 New Hampshire Avenue, Silver Spring, MD 20993 USA

**Keywords:** generic, ocular PBPK, ophthalmic solution, PBPK

## Abstract

**Background:**

The development of generic ophthalmic drug products is challenging due to the complexity of the ocular system, and a lack of sensitive testing to evaluate the interplay of physiology with ophthalmic formulations. While measurements of drug concentration at the site of action in humans are typically sparse, these measurements are more easily obtained in rabbits. The purpose of this study is to demonstrate the utility of an ocular physiologically based pharmacokinetic (PBPK) model for translation of ocular exposure from rabbit to human.

**Method:**

The Ocular Compartmental Absorption and Transit (OCAT™) model within GastroPlus® v9.8.2 was used to build PBPK models for levofloxacin (Lev), moxifloxacin (Mox), and gatifloxacin (Gat) ophthalmic solutions. in the rabbit eye. The models were subsequently used to predict Lev, Mox, and Gat exposure after ocular solution administrations in humans. Drug-specific parameters were used as fitted and validated in the rabbit OCAT model. The physiological parameters were scaled to match human ocular physiology.

**Results:**

OCAT model simulations for rabbit well described the observed concentrations in the eye compartments following Lev, Mox, and Gat solution administrations of different doses and various administration schedules. The clinical ocular exposure following ocular administration of Lev, Mox, and Gat solutions at different doses and various administration schedules was well predicted.

**Conclusion:**

Even though additional case studies for different types of active pharmaceutical ingredients (APIs) and formulations will be needed, the current study represents an important step in the validation of the extrapolation method to predict human ocular exposure for ophthalmic drug products using PBPK models.

**Supplementary Information:**

The online version contains supplementary material available at 10.1007/s11095-022-03390-z.

## Introduction

Ophthalmic drug products are used to treat local ocular diseases in humans such as glaucoma, inflammation, or infection. The development of ophthalmic generic drug products faces significant challenges as the active pharmaceutical ingredients (APIs) reach the site of action before they enter the systemic circulation and usually produce very low to undetectable levels in the systemic circulation. In addition, in silico analysis demonstrated that systemic pharmacokinetic (PK) metrics sensitivity to changes in formulations’ physicochemical properties are not correlated to local PK metrics modifications [1]. Hence, the traditional bioequivalence (BE) approaches based on systemic drug concentrations for orally administered drugs are typically not applicable [2]. The U.S. Food and Drug Administration (FDA) has ongoing efforts to encourage generic drug development for locally acting drug products. The internal and external research initiatives sponsored by the FDA aim to either develop and validate new tools capable of supporting the development and regulatory assessments of ophthalmic generic drug products, or to enhance the scientific understanding of the interplay between a formulation and the ocular surface [3]. These research programs supported the approval of the first generic product for Restasis^©^ (cyclosporine ophthalmic emulsion) 0.05% single-use vials (eye drops) allowing millions of Americans who suffer from dry eye disease to have access to this treatment.

The development of generic ophthalmic drug products is challenging due to the complexity of the ocular system and a lack of sensitive testing to evaluate its interplay with ophthalmic formulations. Approaches including a combination of *in vitro* characterization, *in vivo* PK, and/or *in vivo* comparative pharmacodynamic (PD)/clinical endpoint BE studies have been recommended by the FDA to demonstrate BE for ophthalmic products. The determination of the appropriate study or combination of studies is based on the complexity of the dosage form (e.g., solution, suspension) and the scientific understanding of drug release and disposition in the eye [4]. However, the associated *in vivo* BE studies impose challenges to the generic industry: high development cost, poor sensitivity to small formulation differences of the PD endpoint, significant variability, and sparse sampling design for the PK study. Those limit the development of ophthalmic generic drugs [5]. Per the Orange Book [6], most of the marketed ophthalmic generics are for ophthalmic solutions with a few others for ophthalmic suspensions. Therefore, gaps are limiting the development and regulatory acceptance of complex ophthalmic formulations. Further research in this area to reduce both developmental and regulatory barriers is a necessity.

Physiologically based pharmacokinetic (PBPK) models were first introduced in the 1970s to support drug product development from preclinical to clinical trials as they can reduce cost and attrition in drug product development [7, 8]. PBPK models are now routinely used by the pharmaceutical industry to predict first-in-human doses for oral formulations based on preclinical data [9], to predict drug-drug interactions based on *in vitro* parameters, or to support formulation optimization based on *in vitro* dissolution data [10]. The numerous applications of PBPK models in the generic industry have been presented elsewhere [11]. Ocular PBPK models can provide an insight into drug partitioning in eye tissues that are not accessible and/or are challenging to sample in humans and serve as an alternative methodology to study ophthalmic drugs PK and PD. The rabbit eye physiology is comparable to human eye physiology [12] and this animal is used as the main preclinical model to investigate the impact of formulation changes on an API ocular exposure. This physiological resemblance makes rabbit the preferred species for PBPK-based extrapolation of human ocular exposure based on preclinical data [12].

In the GDUFA science and research priority initiatives for the fiscal year 2022, research on complex routes of delivery is listed as one of the top priorities. Furthermore, enhancement of PBPK models for these complex routes of delivery to allow their use in supporting alternative BE approaches is identified as a key priority [13]. This is a continuation of a goal established during the first GDUFA period from 2012 to 2017. Starting in 2014, the FDA collaborated with Simulation Plus, Inc. to enhance and validate a state-of-the-art ocular PBPK model. As a result, the Ocular Compartmental Absorption and Transit (OCAT™) model within GastroPlus® had been expanded and validated for the simulation of ophthalmic suspensions and ointments in rabbit [1, 14, 15]. Since 2020, through grant 1U01FD006927-01, the Office of Generic Drugs in the FDA has collaborated with Simulation Plus, Inc. to further advance the ocular PBPK by expanding the existing knowledge base for ocular drug absorption and disposition. This collaboration aims to validate the ability of the OCAT model to predict ophthalmic clinical PK/PD based on PBPK models validated against preclinical data.

To achieve this goal, ophthalmic solutions were the first to be studied. Those are the simplest formulation as the API is fully dissolved in the dosage form before application. PK-PD relationships can be nonlinear; therefore, before performing a clinical prediction solely based on PD data, it must be proven that the OCAT model can accurately extrapolate clinical PK exposure. Therefore, this article describes the use of the OCAT model to predict clinical ocular tissues’ exposure once it has been validated using ocular rabbit PK data.

Levofloxacin (Lev), moxifloxacin (Mox), and gatifloxacin (Gat) are fluoroquinolones administered as ophthalmic solutions to treat local infections on the surface of the eye. The abundance of published data on systemic and local exposures in rabbit and human makes these drugs ideal test cases for the first step of this research.

This study includes: 1) the development and validation of systemic PBPK models for Lev, Mox, and Gat; 2) the development and validation of the ocular PBPK models for the three APIs in rabbits and 3) the prediction of clinical ocular exposure of Lev, Mox, and Gat using the validated rabbit PBPK models.

## Methods

### Software and model structure

GastroPlus^©^ (version 9.8.2 Simulation Plus Inc., Lancaster, CA, USA) was used for computer simulation of Lev, Mox, and Gat biodistribution in rabbit and human ocular tissues. For each API, the model structure integrates a PBPK model reflecting systemic tissue distribution and clearance, an OCAT model (Version 3) describing ocular drug absorption and disposition, and an Advanced Compartmental Absorption & Transit (ACAT™) model to capture intestinal absorption of the portion of the dose that entered intestine through nasolacrimal drainage and swallowing. Input parameters for all compounds were either obtained from literature or fitted based on *in vivo* data. All API-specific and formulation-specific parameters are presented in Table I. The details of the OCAT model describing ocular drug absorption and disposition have been presented in previous publications [1, 15].

**Table I:** Parameter Values Implemented in the OCAT-PBPK Model for Levofloxacin, Moxifloxacin, and Gatifloxacin

Parameter	Definition	Units	Levofloxacin		Moxifloxacin		Gatifloxacin	
*Physicochemical Properties*			*Value*	*Source*	*Value*	*Source*	*Value*	*Source*
MWt	molecular weight	g/mol	361.4	AP10*	401.44	AP10*	375.4	AP10*
logP(neutral)	Log octanol/water partition coefficient	-	-0.4	[16]	0.01	[17]	-0.8	[18]
F_u_	Plasma unbound percent	%	75	[19]	84	[20]	80	[21]
F_u melanin_	Percent unbound to melanin	%	0.1	fitted	1	fitted	0.4	fitted
R_bp_	Blood to plasma concentration ratio		0.95/0.84**	fitted	0.9	fitted	0.9	fitted
Solubility	Maximum amount dissolved in water	mg/mL	50 (pH 6.9)	[16]	17.68 (pH 7.4)	[22]	60 (pH 4)	[23]
pKa	Acidity constant	-	5.73/6.8/8.08	[16]	1.53/6/9.2	AP10	1.04/5.92/9.1	AP10
			Acid/Base/Base		Base/Acid/Base		Base/Acid/Base	
Peff	Intestinal permeability	× 10^–4^ cm/s	3.97	[24]	0.1/4**	fitted	2	fitted
*Clearance*								
Renal_fraction_	Rabbit/Human renal clearance as a fraction of kidney blood flow	-	0.1/0.09**	fitted	0.11/0.02**	fitted	0.12	fitted
CLint	Rabbit/Human liver intrinsic clearance	L/h	0.054/ 2**	fitted	3.7/8**	fitted	0.16/2.7**	fitted
OCAT™ *parameters*								
Perm_Cornea_epi_	Cornea epithelium permeability	× 10^–7^ cm/s	1.85	fitted	8	fitted	3	fitted
Perm_Cornea_str_	Cornea stroma permeability	× 10^–5^ cm/s	1.04	GP***	1.43	GP	7.78	GP
Perm_Conjunctiva_	Conjunctiva permeability	× 10^–7^ cm/s	2.5	fitted	4.36	GP	4.35	GP
Perm_AH_	Aqueous humor permeability	× 10^–6^ cm/s	9.14	GP	40	fitted	8.76	GP
Perm_ICB_	Iris-Ciliary Body permeability	× 10^–5^ cm/s	8.98	GP	64	GP	8.94	GP
Perm_Sclera_	Sclera permeability	× 10^–6^ cm/s	5.71	GP	7.89	GP	4.28	GP
Perm_Choroid_	Choroid permeability	× 10^–4^ cm/s	1.02	GP	1.42	GP	7.68	GP
Perm_Retina_	Retina permeability	× 10^–6^ cm/s	2.01	GP	14.3	GP	2	GP
Perm_V.H_	Vitreous Humor permeability	× 10^–6^ cm/s	7.2	GP	6.7	GP	6.9	GP
SAR_Choroid_	Choroid systemic absorption rate	× 10^–4^ s^−1^	1.53	GP	2.12	GP	1.15	GP
SAR_Retina_	Retina systemic absorption rate	× 10^–4^ s^−1^	1.4	GP	9.95	GP	1.39	GP
SAR_Conjunctiva_	Conjunctiva systemic absorption rate	× 10^–4^ s^−1^	3.77	GP	3.78	GP	3.77	GP
SAR_ICB_	Iris-Ciliary Body systemic absorption rate	× 10^–3^ s^−1^	1.25	fitted	0.83	GP	0.7	fitted

*: AP10 refers to ADMET Predictor^®^ module version 10

**: Parameter values for rabbit and human respectively

***: GP refers to GastroPlus^®^ default parameters

### Systemic PBPK models

For all fluoroquinolones, published preclinical and clinical studies used to develop and validate the systemic PBPK model are listed in supplementary material 1. For all APIs, a PBPK model with all tissues defined as perfusion limited was selected. Tissue/plasma partition coefficients (Kps) were calculated using the default Lukacova method for the perfusion-limited tissues[25]. The blood to plasma ratio (Rbp) was adjusted so the predicted volume of distribution (Vd) corresponds to the calculated one based on non-compartmental analysis (NCA) of intravenous (IV) data. Renal clearance was parametrized as a fraction of kidney blood flow. These fractions were fitted to describe the total renal excretion of Lev, Mox, and Gat [21, 26, 27]. Liver clearance was then fitted to capture the observed IV data for each API. Mox and Gat observed data following oral administration were captured by adjusting the intestinal permeability (Peff). For Lev, the Peff value estimated by ADMET Predictor was able to describe the observed oral data. If necessary, different Rbp, fraction of kidney blood flow, liver clearance, and gut permeability values were defined for rabbit and human to account for inter-species differences and describe observed PK data in both species. Additional details about the parameterization of the systemic PBPK models are presented in supplementary material 1.

### Ophthalmic PK clinical extrapolation strategy

Published preclinical and clinical studies used to perform the clinical PK extrapolation are listed in supplementary material 2. To predict human ophthalmic exposure following fluoroquinolone solution administration, the OCAT model was first developed and validated in rabbit. Preclinical data describing the ocular exposure in albino New Zealand white (NZ) rabbits from multiple studies were available for each API. One or two studies were used to develop the baseline OCAT model for each API. The initial simulation was performed for each API with all default OCAT parameters, and the predictions were compared with observed data from selected studies in NZ rabbits. Mispredictions were addressed by modifying/fitting a minimum number of API-specific OCAT parameters for each compound. The model was deemed acceptable if the simulated ocular tissues’ concentration time courses could be overlaid with observed data, and if no systematic mispredictions could be identified. The fitted parameters were validated by predicting ocular PK for additional studies in NZ rabbits. The baseline model was subsequently extended to include melanin binding by fitting the fraction bound to melanin to describe the iris-ciliary body (ICB) concentration–time course of each API in pigmented Dutch Belted (DB) rabbits. External validation of melanin binding was performed, when possible, based on the availability of published data. The same melanin binding fraction was used to describe melanin binding in the remaining melanin-containing tissues (sclera, retina, choroid) for which observed data were not available.

**Fig. 1:**
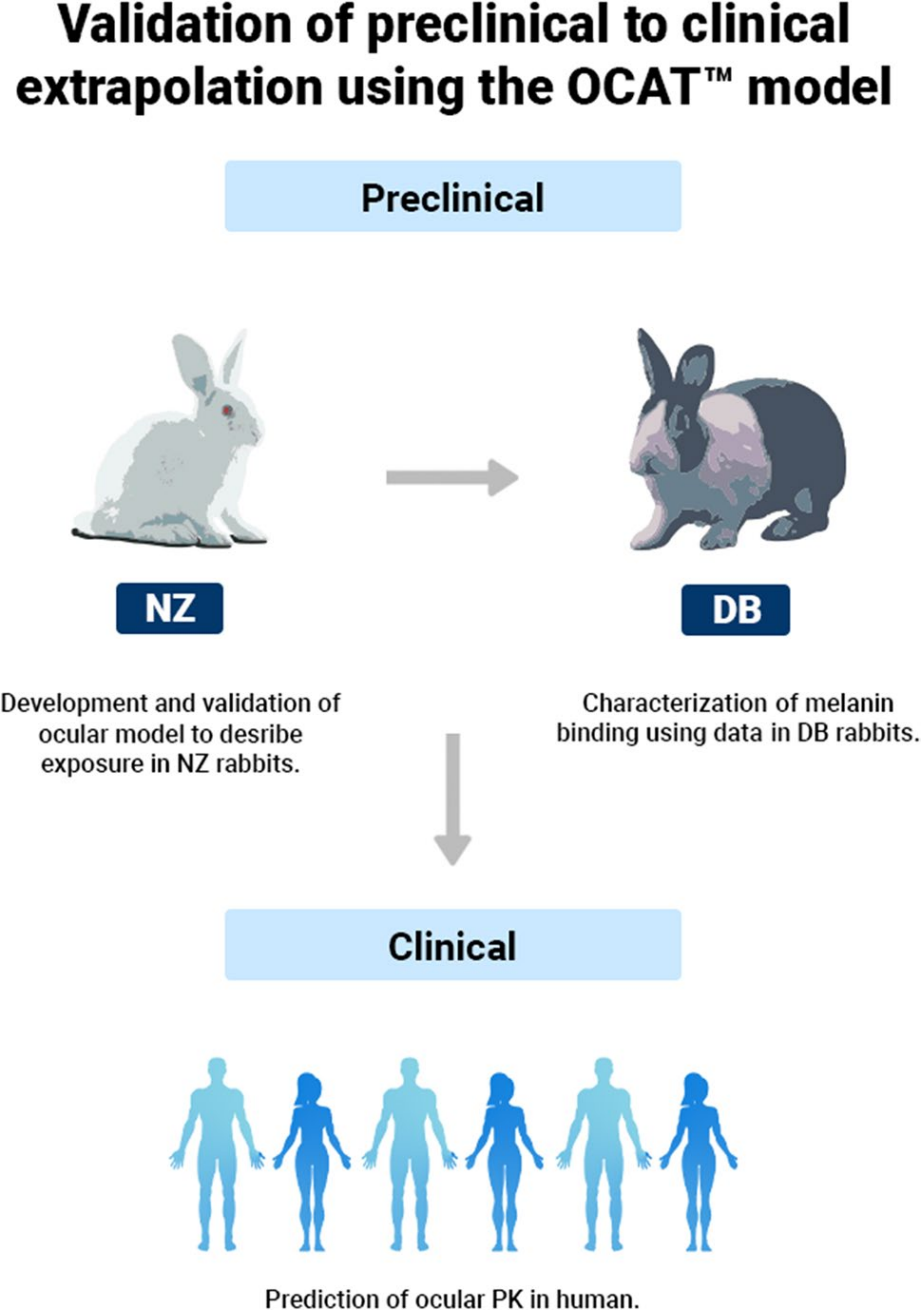
PBPK-based strategy to perform and validate the preclinical to clinical ophthalmic PK extrapolation using the OCAT model. Abbreviations: NZ: New Zealand; DB: Dutch Belted; EV: external validation.

The validated models integrating melanin binding were used to predict the ocular PK in human. API-specific parameters remained at the same values as fitted against rabbit ocular PK data. The physiological parameters were adjusted to match human ocular physiology. The dose, dose volume, and dose administration schedule were set according to clinical studies. Simulated human ocular PK profiles were compared with observed concentration data to assess the OCAT models’ ability to predict human ocular exposure based on preclinical data. A summary of the preclinical to clinical extrapolation strategy is presented in Fig. 1.

### Parameterization of OCAT models

The default ocular physiologies for NZ and DB rabbits available in GastroPlus version 9.8.2 were used for model development and validation. As described above, rabbit-to-human extrapolation was then performed by switching the DB rabbit physiology to the default human ocular physiology available in GastroPlus version 9.8.2. For all studies, the dose administered volume, and dosing schedules were adjusted based on the information reported in the literature.

The drainage rate constant was set to 1 min^−1^, which is the default value, for all species as formulations do not present excipients affecting their viscosities. Therefore, the formulation viscosity is expected to be comparable to water. The pre-cornea maximum volume was set to 35 and 37 µL for rabbit and human physiologies, respectively, to account for the physiological tear volume (5 or ≈ 7 µL) plus the typically administered volume of fluoroquinolone solutions (30 µL). Therefore, if a volume superior to 30 µL is administered in a preclinical/clinical study, the OCAT model automatically discards the excess fluid volume. During simulation, following the solution eye drop administration, the volume of the pre-cornea compartment gradually decreased to the physiological tear volume through nasolacrimal drainage. This process clears the drug dissolved in the pre-cornea compartment before its absorption in the cornea or conjunctiva. The OCAT model assumes that evaporation and tear fluid absorption is negligible.

## Results

### Systemic PBPK models

PBPK models describing intestinal absorption and systemic disposition of Lev, Mox, and Gat in rabbits and human were developed and validated using plasma concentration time profiles following IV and/or oral administration. In summary, for Lev, Mox, and Gat, settings for Kp calculation were calibrated to match the reported Vd after IV administration. Elimination was modeled by a combination of renal excretion (defined as the fraction of kidney blood flow) and linear liver clearance and was parameterized to capture total observed clearance after IV administration as well as the unchanged API amount excreted in the urine. For the three APIs, their respective PBPK models accurately described the observed data. Details of model development and results obtained for each fluoroquinolone are presented in supplementary material 1.

### Rabbit OCAT Models

#### Levofloxacin

**Fig. 2:**
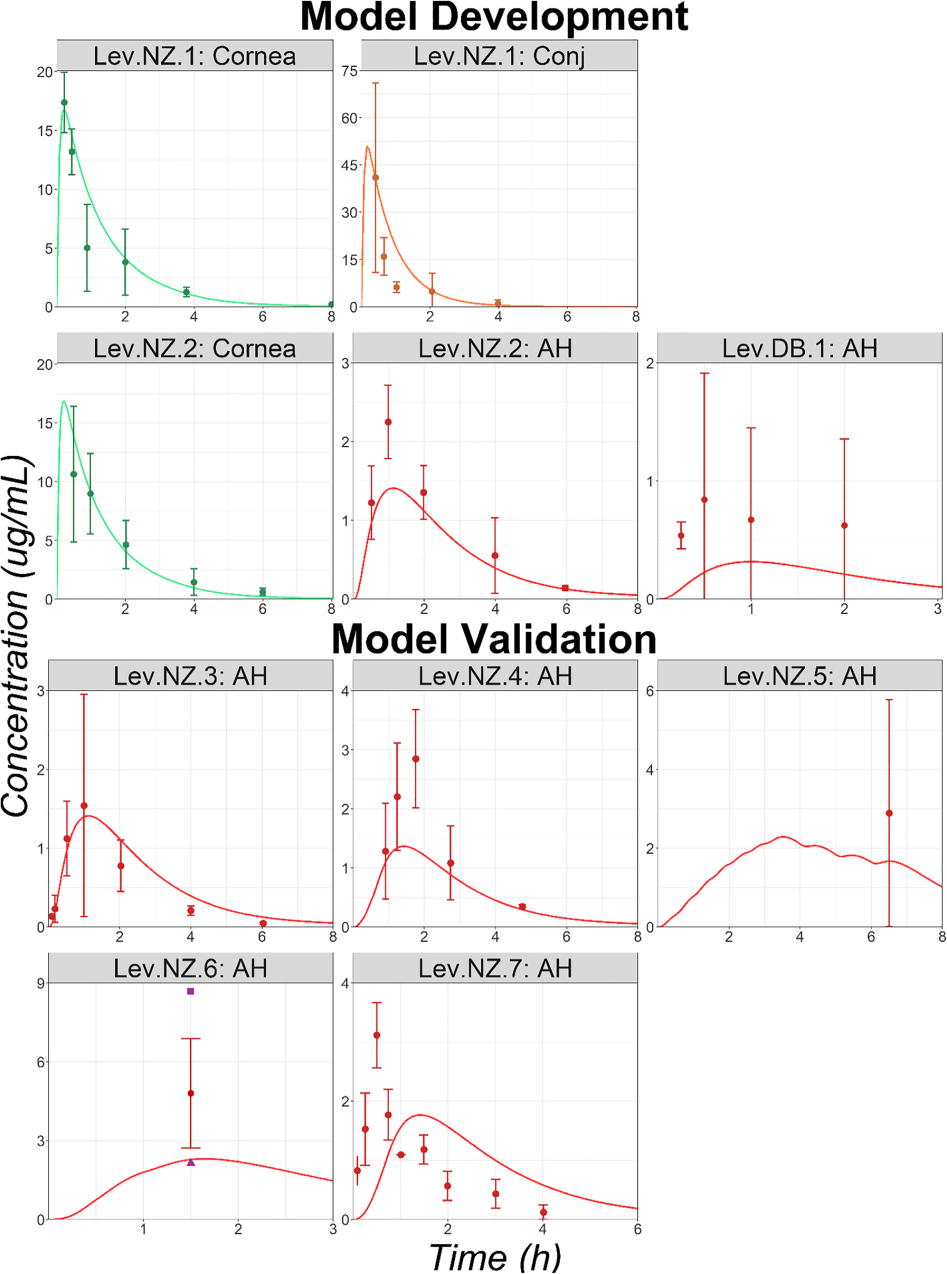
Ocular Time Courses in NZ and DB Rabbits Following a Single or Multiple Topical Administration of Levofloxacin Solution (Studies Lev.NZ.1 to Lev.NZ.7 and Lev.DB.1). Abbreviations: AH: Aqueous Humor; Conj: Conjunctiva. Observed mean concentrations and corresponding standard deviations are represented by circles and error bars, respectively. Purple squares and triangles represent the reported study-specific maximum and minimum observed concentrations, respectively.

Seven preclinical studies in NZ rabbits and one in DB rabbits with reported Lev ocular tissue concentrations were identified in the literature (studies codes: Lev.NZ.1 to Lev.NZ.7 and Lev.DB.1; data sources and study protocols are listed in supplementary material 2). Lev.NZ.1, which lists cornea and conjunctiva concentrations, and Lev.NZ.2, which lists cornea and aqueous humor (AH) concentrations, were used for model development. The cornea epithelium and conjunctiva permeabilities were fitted to final values of 1.85*E-7 and 2.5*E-7 cm/s, with permeabilities for all remaining ocular tissues kept at default values (estimated by GastroPlus from the API’s physicochemical properties). The ICB systemic absorption rate constant was also adjusted to a final value of 1.246*E-3 1/s. The model reasonably captures the observed cornea, conjunctiva, and AH data for NZ rabbit (Fig. 2). The fitted parameters were validated by predicting the ocular concentration time courses following the administration of a Lev solution to NZ rabbits for different strength and dosing regimens (studies: Lev.NZ.3 to Lev.NZ.7). Final simulations for AH concentration time courses are presented in Fig. 2. AH concentrations following single and multiple dose administrations of Lev solution at different strengths are well predicted. Hence, the external validation was completed, and the model was deemed acceptable for further analysis. To describe the ocular concentration time courses following the administration of a Lev solution to DB rabbits (Lev.DB.1), the unbound fraction to melanin for ICB was fitted to 0.1%, and all other ocular parameters were kept at the same values as validated for studies in NZ rabbits. The same value was used for retina, choroid, and sclera melanin binding. The simulated and observed AH concentrations for study Lev.DB.1 are presented in Fig. 2. Description of the observed ICB data by the OCAT model is presented in supplementary material 3. Overall, for Lev, the tissue concentration time courses of the anterior segment of the eye are reasonably well described by the OCAT model.

#### Moxifloxacin

**Fig. 3:**
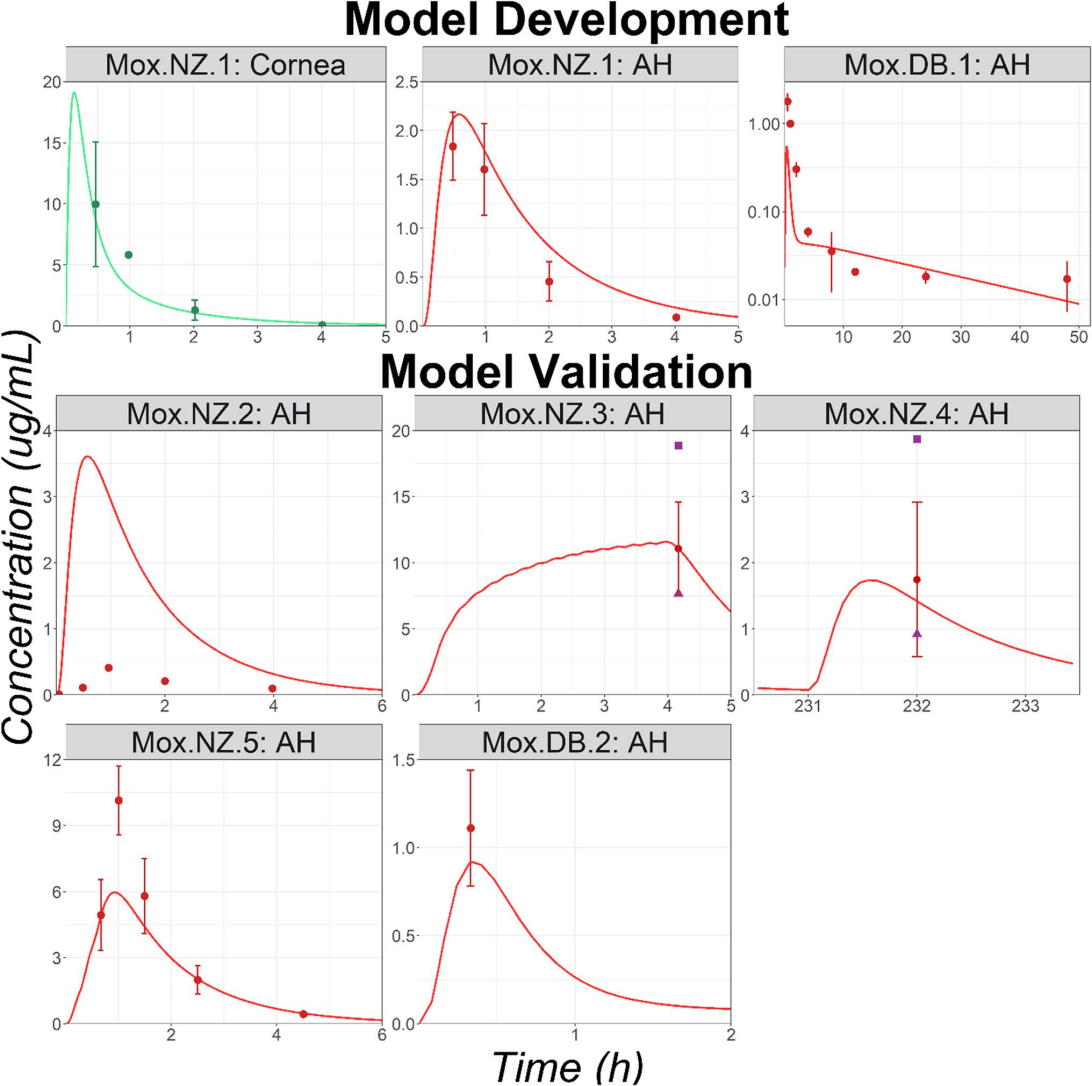
Ocular Time Courses in NZ and DB Rabbits Following a Single or Multiple Topical Administration of Moxifloxacin Solution (Studies Mox.NZ.1 to Mox.NZ.5 and Mox.DB.1 to Mox.DB.2). Abbreviations: AH: Aqueous Humor. Observed mean concentrations and corresponding standard deviations are represented by circles and error bars, respectively. Purple squares and triangles represent the reported study-specific maximum and minimum observed concentrations, respectively.

Five preclinical studies in NZ rabbits and two in DB rabbits with reported Mox ocular tissue concentrations were identified in the literature (studies codes: Mox.NZ.1 to Mox.NZ.5 and Mox.DB.1 to Mox.DB.2; sources and study protocols are listed in supplementary material 2). Mox.NZ.1, which lists cornea and AH concentrations, was used for model development. Cornea epithelium and AH permeabilities were set to 8*E-7 and 4*E-5 cm/s respectively, with permeabilities for all remaining ocular tissues kept at default values (estimated by GastroPlus from API’s physicochemical properties). The model could describe the observed data following a single administration of Mox 0.5% (Mox.NZ.1, Fig. 3). The model was then validated by predicting AH exposure from four additional studies performed in NZ rabbits (studies: Mox.NZ.2 to Mox.NZ.5). The model adequately described the observed AH data following multiple dose administrations of Mox 0.5% (studies: Mox.NZ.3 to Mox.NZ.5, Fig. 3). However, AH concentration following the single dose administration of Mox 0.5% was largely overpredicted for the study Mox.NZ.2 (Fig. 3). Maximum AH concentration (Cmax) in study Mox.NZ.2 is around four-fold lower than the one observed in study Mox.NZ.1, despite few differences in the preclinical protocol. This shows a significant interstudy variability which had already been observed for other APIs [28]. Unbound fraction to melanin in ICB, was fitted to 1% based on preclinical studies in DB rabbits. The same value was used for retina, choroid, and sclera melanin binding. The observed AH concentrations in DB rabbits are well predicted for the Mox.DB.2 study and underpredicted for the Mox.DB.1 study (Fig. 3). Description of the observed ICB (study Mox.DB.1) data by the OCAT model are presented in supplementary material 3. Those observed data are also underpredicted but the observed and simulated ratios of Cmax and exposure between the AH and ICB tissues are comparable. In study Mox.DB.1, the cornea data were also underpredicted (data not shown). Modification of tear elimination pathway parameters would help to describe all observed concentrations provided in this study. However, as this research aims to use the default physiologies, a specific fit of a physiological parameters was excluded, and the model results were accepted as such. Additionally, Mox.DB.2 presented observed conjunctiva data. Their good description by the model is presented in supplementary material 3. In summary, considering the significant interstudy variability in the observed data, the OCAT model adequately described the observed ocular tissues concentrations time courses.

#### Gatifloxacin

**Fig. 4:**
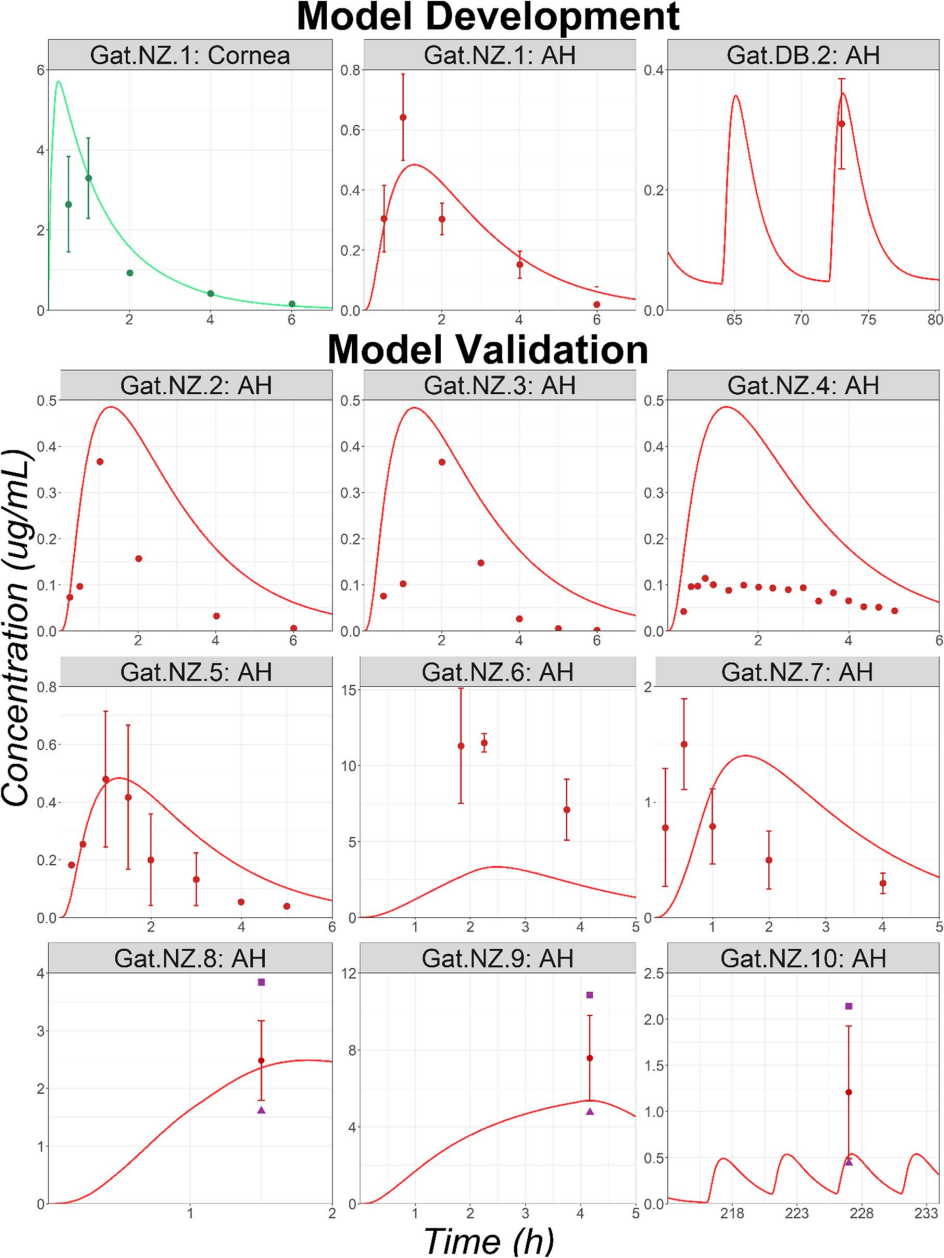
Ocular Time Courses in NZ and DB Rabbits Following a Single or Multiple Topical Administration of Moxifloxacin Solution (Studies Gat.NZ.1 to Gat.NZ.9 and Gat.DB.2). Abbreviations: AH: Aqueous Humor. Observed mean concentrations and corresponding standard deviations are represented by circles and error bars, respectively. Purple squares and triangles represent the reported study-specific maximum and minimum observed concentrations, respectively.

Ten preclinical studies in NZ rabbits and two in DB rabbits with reported Gat ocular tissue concentrations were identified in the literature (studies codes: Gat.NZ.1 to Gat.NZ.10 and Gat.DB.1 to Gat.DB.2; sources and study protocols are listed in supplementary material 2). Gat.NZ.1, which lists cornea and AH concentrations, was used for model development. Cornea epithelium permeability was set to 3*E-7 cm/s and the ICB systemic absorption rate was fitted to 7*E-4 1/s to describe the AH elimination slope. All other OCAT model parameters were kept at default values (estimated by GastroPlus from the API’s physicochemical properties). The model could describe the observed data following single administration of Gat 0.3% (Mox.NZ.1, Fig. 4). This model was validated by describing the observed ophthalmic concentrations in the remaining studies performed on NZ rabbits. The model parameters for the dose and dosing regimen were adjusted to match the information presented in the literature for each study. Overall, the model adequately described the observed AH data, except for studies Gat.NZ.4 and Gat.NZ.6, which were over- and under-predicted, respectively (Fig. 4). The unbound fraction to melanin parameter for ICB was fitted to 1% based on ICB data provided in study Gat.DB.2 (results presented in supplementary material 3). The same value was used for retina, choroid, and sclera melanin binding. The conjunctiva concentration time course following the single administration of Gat 0.3% solution was well described (study Gat.DB.1, results presented in supplementary material 3).

#### Ophthalmic PK clinical extrapolation

The clinical extrapolations were performed using the validated OCAT drug-specific parameters from rabbit simulations. The physiological parameters were adjusted to match human ocular physiology. In addition, the dose, dose volume, and dosing regimen were parameterized to match the published information for each human trial. Clinical data were obtained from patients undergoing either cataract, keratoplasty, or vitrectomy surgeries. Additionally, conjunctival biopsies in healthy subjects were available. Based on the surgical procedure, sampling from different ocular tissues is performed: AH for cataract and cornea and AH for keratoplasty; AH and VH for vitrectomy; and conjunctiva in healthy subject conjunctival biopsies. The sources, study protocols, and disease conditions for all clinical studies are listed in supplementary material 2. To assess the ability of the OCAT model to predict human ocular exposure, individual simulations were performed for each study, and results were compared with the corresponding observed data. Most of the published clinical studies were performed on patients. At this stage, the disease conditions were not accounted for in the ophthalmic physiology. To understand if the disease condition could impact the in silico extrapolation, clinical studies were pooled based on the type of surgery performed.

#### Cataract Surgery

**Fig. 5:**
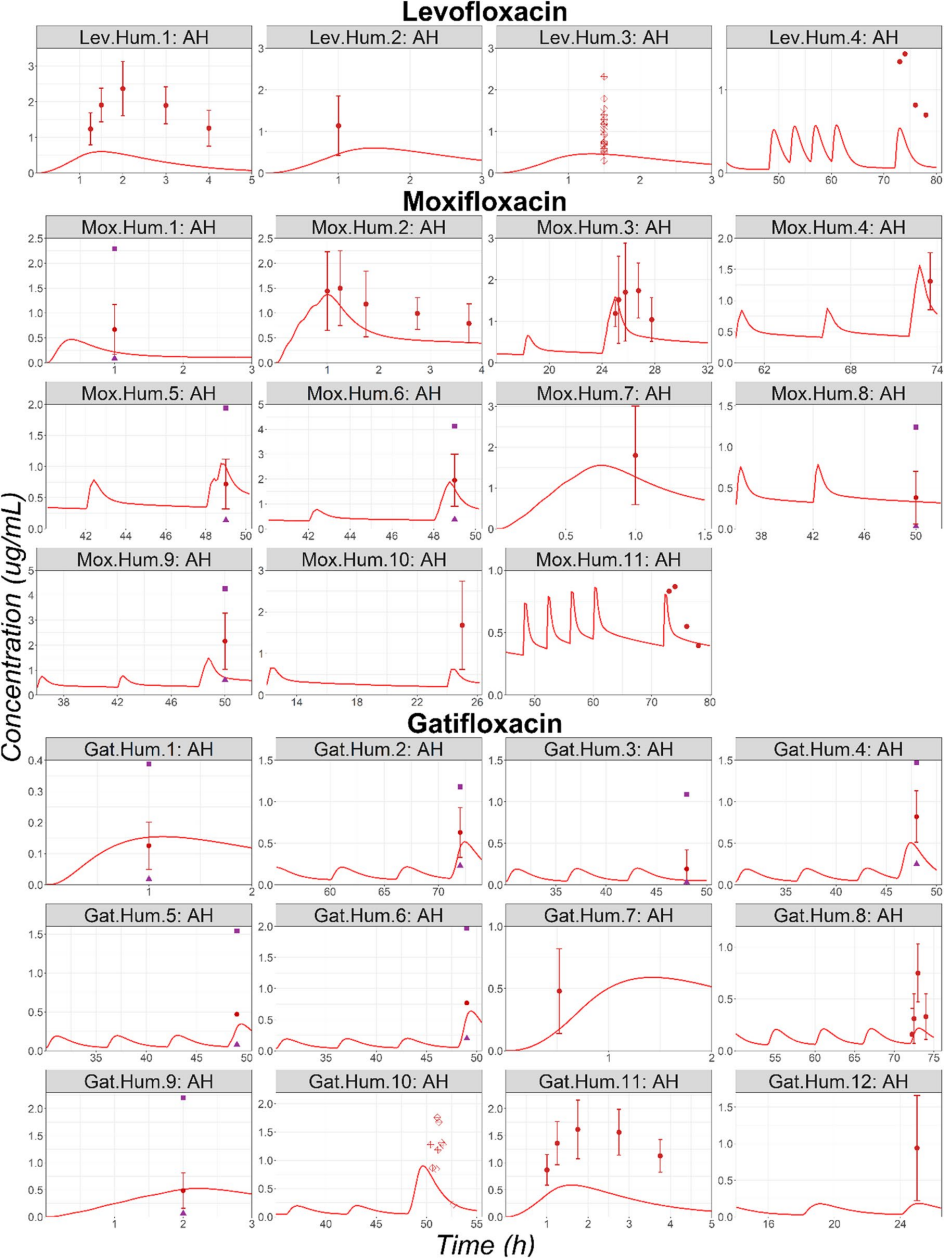
AH Time Courses in Human Undergoing Cataract Surgery Following a Single or Multiple Topical Administration of Levofloxacin, Moxifloxacin, or Gatifloxacin Solution. Abbreviations: AH: Aqueous Humor. Observed mean concentrations and corresponding standard deviations are represented by circles and error bars, respectively. Purple squares and triangles represent the reported study-specific maximum and minimum observed concentrations, respectively. For studies Lev.Hum.3 and Gat.Hum.10, diamonds represent individual subject concentrations.

Observed AH concentrations for 4, 11, and 12 studies were obtained in literature for Lev, Mox, and Gat, respectively. For most of the studies, the simulated profiles adequately describe the observed data (Fig. 5). Observed data were significantly underpredicted for studies Lev.Hum.1 and Gat.Hum.11. Interestingly, the observed data for these two studies were obtained from the same publication. No specific characteristics were identified in the clinical protocol that could explain the model underprediction for both Lev and Gat. This example clearly demonstrates the presence of interstudy variabilities. Overall, the observed data following the single or multiple administration of Lev, Mox, or Gat as ophthalmic solutions are well predicted demonstrating the ability of the OCAT model to predict AH exposure in cataract patients using the OCAT model previously validated based on rabbit PK studies.

#### Keratoplasty Surgery

**Fig. 6:**
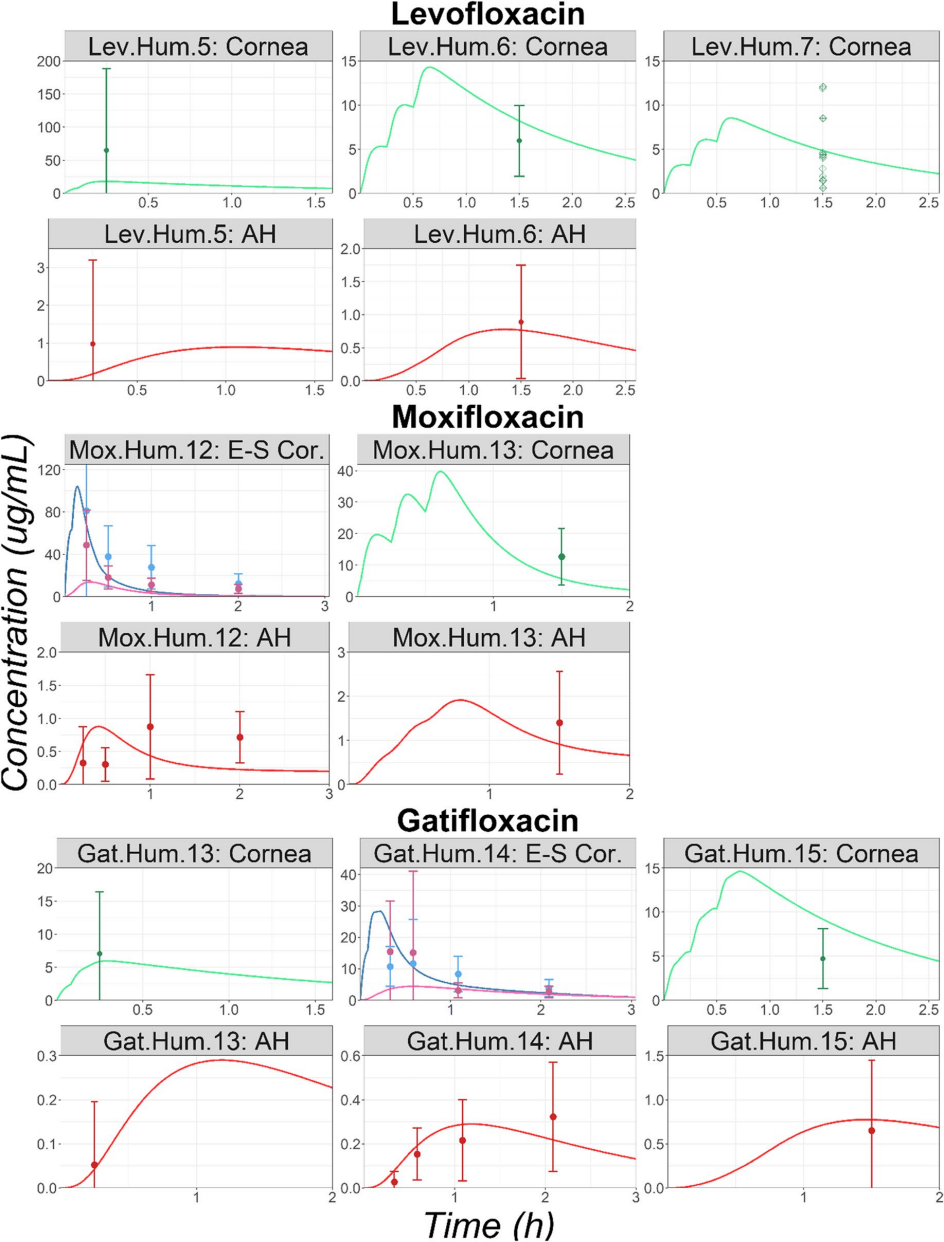
Time Courses in Human Undergoing Keratoplasty Surgery Following Multiple Topical Administration of Levofloxacin, Moxifloxacin, or Gatifloxacin Solution. For the studies Max.Hum,12 and Gat.Hum.14, the blue line and dots represent the corneal epithelium, and the pink line and dots represent the corneal stroma. Abbreviations: AH: Aqueous Humor; E-S Cor.: Corneal Epithelium and Stroma. Observed mean concentrations and corresponding standard deviations are represented by circles and error bars, respectively. For studies Lev.Hum.7 diamonds represent individual subject concentrations.

Observed cornea and AH concentrations for three, two, and three studies were obtained in literature for Lev, Mox, and Gat, respectively. Overall, the models well described both the cornea and AH observed concentrations following multiple topical dose administrations of each API to patients undergoing keratoplasty surgery (Fig. 6). In addition, for study Mox.Hum.12 and Gat.Hum.14, the model accurately described the observed cornea epithelium and corneal stroma concentration profiles.

#### Vitrectomy Surgery

**Fig. 7:**
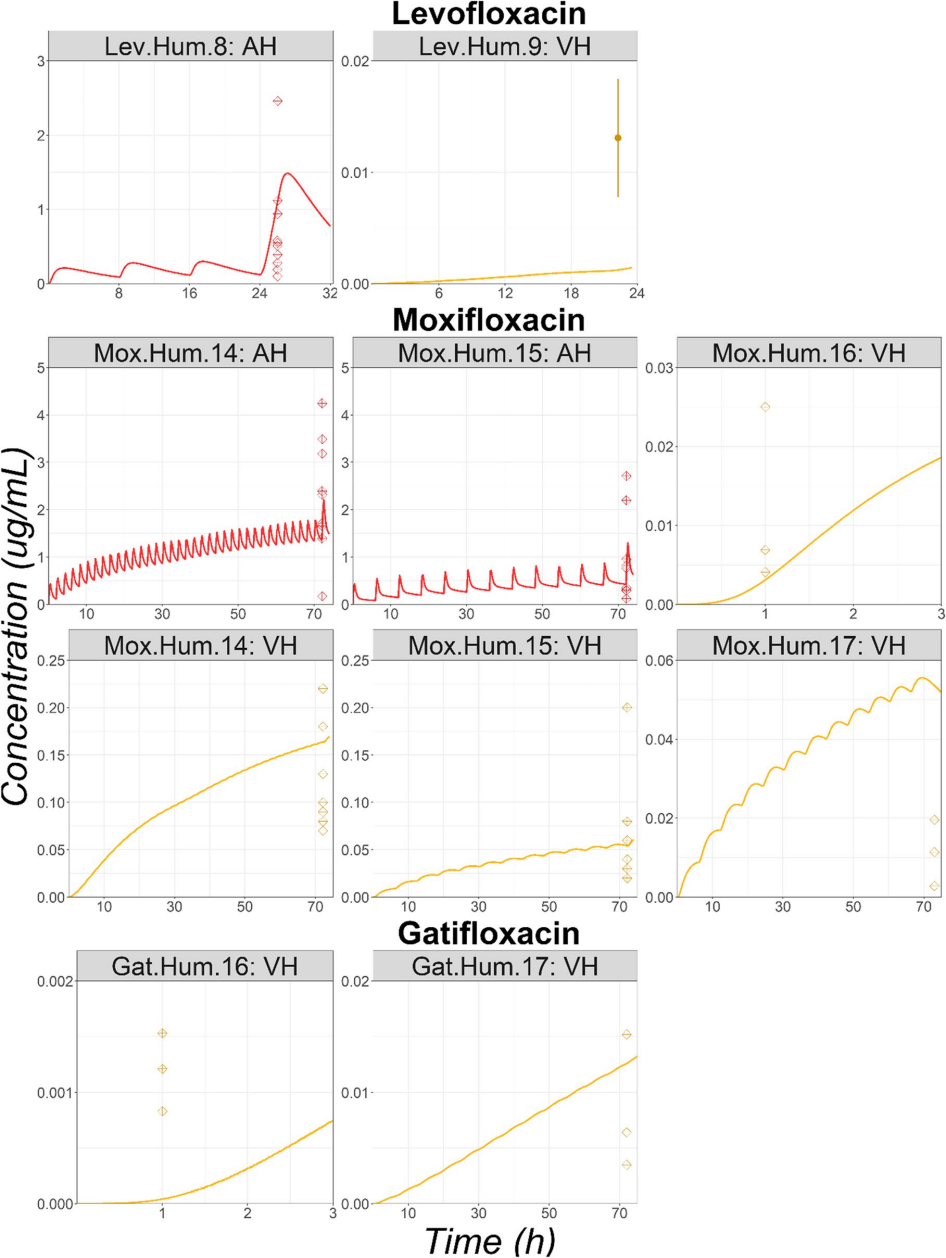
Observed (squares) and Simulated (lines) Ocular Time Courses in Human Undergoing Vitrectomy Surgery Following Multiple Topical Administration of Levofloxacin, Moxifloxacin, or Gatifloxacin Solution. Abbreviations: AH: Aqueous Humor; VH: Vitreous Humor. Observed individual subject concentrations are represented by diamonds. For study Lev.Hum.9, the mean concentration and standard deviation are represented by a circle and error bars.

Observed AH and VH concentrations for two, four, and two studies were obtained in literature for Lev, Mox, and Gat, respectively. Overall, the AH data are relatively well predicted by the model (Fig. 7). For VH, the extrapolation results are sparse (Fig. 7). Lev VH concentrations following multiple dose administrations were underpredicted (Lev.Hum.9). However, for Mox, VH concentrations following multiple dose administrations were well predicted for three studies (Mox.Hum.14, Mox.Hum.15, Mox.Hum.17) and overpredicted for one study (Mox.Hum.16). For Gat, VH concentrations were well predicted following the administration of Gat 0.3% four times daily for 3 days (Gat.Hum.17), whereas the observed data following three administration every fifteen minutes for a total of 3 doses were unpredicted (Gat.Hum.16). In summary, the OCAT model adequately described human AH observed concentrations in patients undergoing vitrectomy surgery. Human VH concentrations were well predicted in half of the studies, but few studies were either under or over-predicted.

#### Healthy Subjects

**Fig. 8:**
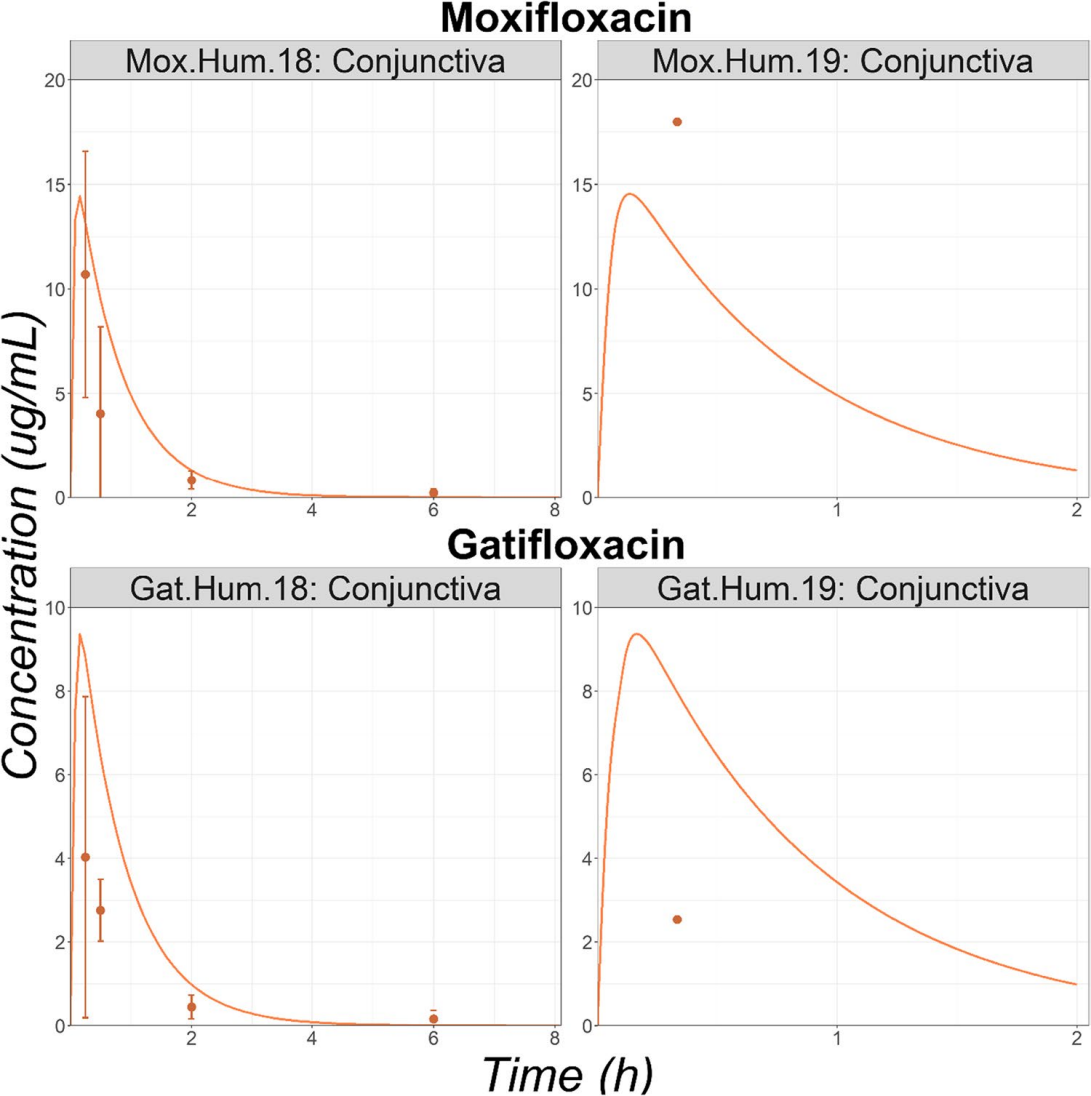
Observed (squares) and Simulated (lines) Conjunctiva Time Courses in Human Undergoing Conjunctiva Biopsies Surgery Following a Single Topical Administration of, Moxifloxacin or Gatifloxacin Solution. Observed mean concentrations and corresponding standard deviations are represented by circles and error bars, respectivel.

Observed conjunctiva concentrations for two studies were obtained from the literature for Mox and Gat. Overall, the OCAT model describes relatively well the observed conjunctiva concentrations following the single administration of either Mox or Gat solution (Fig. 8). Only the Gat.Hum.19 study is overpredicted. For Gat, there is a significant inter-study variability as the study Gat.Hum.18 reported a conjunctiva concentration of 4.03 µg/mL (CV% = 95.3), 15 min post administration, whereas the Gat.Hum.19 study reported a concentration of 2.54 µg/mL, 20 min after the administration. However, even by factoring the interstudy variability, the single observed data point of study Gat.Hum.19 is overpredicted.

## Discussion

An understanding of the ocular absorption mechanism is necessary for both the pharmaceutical industry and the regulatory agencies to support the development and evaluation of new and generic drug products. A previous research project described the methodology to develop and validate an OCAT model for a rabbit physiology [1]. That project was focused solely on rabbit data and was a proof of concept of the benefits of in silico approaches to support the development of ophthalmic drug products. The current study builds upon the previous project and demonstrates the ability of the OCAT model to predict human ocular exposure. This is achieved by performing PK extrapolation using a validated PBPK model based on rabbit data for three APIs formulated as topical ophthalmic solutions. Additionally, this study proposes a strategy to perform model-based clinical extrapolation for ophthalmic solutions.

To perform the clinical extrapolation, the drug-specific parameters of the OCAT model were kept constant between the DB rabbit and human. Only the physiological parameters were changed between the two species. With these settings, the human mean ocular concentrations were well predicted for the three APIs. This indicates that the APIs’ ocular permeability parameters are likely to be the same *in vivo* for rabbit and human. However, due to significant interstudy variability, some of the clinical data could not be captured by the model. During this research project, some factors that could help to refine the predictions and explain part of the interstudy variability were not included in the model due to the lack of details in published studies. In most cases, the observed data in human are sampled during ophthalmic surgeries. Even though the type of surgery did not seem to impact the ocular distribution in general, the treatments that are administered preoperatively could influence the interplay between a drug product and the eye surface. Topical antisepsis [29], topical or regional anesthesia [30], and topical antibiotics [31] are used together or in sequence to avoid pain during surgery and post-operative complications. These drugs are typically administered topically in the days and hours that precede the surgery for the antibiotics, the hour before the surgery for the anesthetics, and right before the incision for the antisepsis. Administration of topical formulations on the surface of the eye could impact the ocular PK of the studied drug by affecting the tear dynamics. First, a mechanical interaction may occur. The tear space can accommodate only a limited volume of fluid [12]. If the time between the administration of multiple topical formulations is not sufficient to clear most or all the first administered formulation, then the second formulation could be impacted by a higher spillage out of the conjunctival sac combined with a higher tear drainage rate. Secondly, the anesthetic drugs xylocaine hydrochloride, proparacaine hydrochloride, and tetracaine hydrochloride were tested in clinical studies to assess their impact on tear dynamics. It was found that proparacaine hydrochloride could reduce the mean tear quantity by up to 35% [32]. Proparacaine hydrochloride demonstrated a reduction of 75% and 66% of the mean tear volume for young and old age groups, respectively [33]. In addition, a reduction of 66% in the tear flow was also measured in both age groups. A 30% decrease in tear production was observed after administration of Lignocaine in 50 subjects [34]. Therefore, it seems that tear production and tear volume may be impacted using an anesthesia agent, and the overall effect seems to be drug-dependent. As clinical ocular PK data were obtained during surgeries, and these surgeries utilize local or regional anesthesia, the effect of tears dynamic on ocular exposure may be an explanation of the observed interstudy variability. To test this hypothesis, a parameter sensitivity analysis was performed for study Gat.Hum.11, which is significantly underpredicted by the OCAT model. A reduction of the tear flow rate between 60 and 80% allows the description of the observed human AH concentrations. Simulation results are presented in supplementary material 3. However, the clinical protocols were not always specified in all the publications and surgical practices have evolved over the last 35 years (the time period when the clinical data used here was published). Therefore, it is expected multiple anesthesiologic agents have been used and could explain part of the interstudy variability. As more experience is gained with new case studies using the well-defined clinical protocol, adjustments of the tear flow rate based on the type of anesthetic used and the time of administration of the preoperative drugs should be feasible in the future.

The current version of the human ocular physiology describes a healthy eye. Yet, a large portion of the observed clinical data is obtained from patients. The impact of disease on exposure of APIs may be a key factor to consider.

Based on the Center for Disease Control and Prevention (CDC), cataract is a clouding of the lens in the eye that affects vision [36]. This disease is multifactorial but aging is known to be the major cause [37]. Age-mediated changes of the ocular physiology are not implemented in the current version of the OCAT model. However, age-dependent changes in the tear proteome [38], the tear function in a normal population [39], and tear composition [40] have been reported. Therefore, the pathophysiology itself only affects the lens, which is a non-vascularized ocular tissue, and this is not expected to have an impact on the extrapolation process. Nevertheless, the age of the patients participating in the clinical trial could impact the predictions as their tear drainage system may present differences to a typical healthy individual. Future work focusing on the age-mediated evolution of the ocular physiology will be needed.

Keratoplasty is a surgical procedure aiming to replace all or part of the cornea with healthy cornea tissue from a donor [41]. It has multiple indications (e.g., keratoconus, Fuch’s endothelial dystrophy) [42] and the underlying physiological cause can touch all layers of the corneal tissue. As cornea, along with conjunctiva, represents the first barrier to topical ocular drugs’ absorption, an alteration of its structure could alter the drug’s ophthalmic absorption. Yet, without including the impact of those pathologies on the cornea structure, the mean corneal and AH concentrations in human were well predicted. Their inclusion in the model would be an interesting addition to the OCAT model to better understand and describe the interpatient variability.

Vitrectomy is a type of surgery to treat various diseases affecting the back of the eye tissues: VH and retina among others. Those diseases should have a limited impact on the absorption processes for topical ophthalmic formulations. Therefore, it is not expected they would interfere with the in silico extrapolation process for the tissues forming the anterior segment of the eye. This could be seen in the results as the human AH concentration in patients undergoing vitrectomy surgeries was well predicted. Nevertheless, for VH concentration predictions, the results were sparse, and some studies were well predicted whereas others were either under or overpredicted. Due to the lack of sufficient quantitative VH concentration time courses, the OCAT model could not be calibrated to describe VH exposure in rabbit. This could explain the mixed results in human predictions. In addition, some of the indications for this surgery such as retinal detachment, vitreous hemorrhage, or a retinal macula could impact the VH dynamic and an API’s PK in this biophase and therefore the quality in silico extrapolation. Similar to keratoplasty, the inclusion of the pathophysiologies in the OCAT model could support the description of the interstudy and interpatient variabilities. However, the indications for vitrectomy were not reported in most publications and therefore this could not be studied further as part of this research project.

One of the critical aspects influencing drugs’ exposure in some ocular tissues is melanin binding. Melanin is present in ICB, retina, sclera, and choroid tissues. Binding may lead to drug accumulation in the pigmented tissues and prolonged drug retention in the melanin-containing cells [43]. Melanin content varies based on the species and the tissue type [44]. For the three APIs studied in this research project, observed data were consistently available only for ICB in DB rabbit. These were used to calibrate the linear binding to melanin by fitting the fraction unbound to this protein in the ICB. Then the same fraction was assumed in the remaining melanin tissues. This assumption could not be validated during this project due to the lack of observed data in those tissues in both species. However, by using this parameter setting, the differences in melanin content between tissues, and the potential impact on the fraction unbound, are not implemented in the model. In addition, using the same fraction unbound between DB rabbit and human assumes the differences in melanin content between species would not impact the fraction unbound. It is unlikely that ICB concentration data would be available in humans due to the impossibility to sample this tissue during an ethical clinical study. Ideally, the melanin binding would be set on observed data in DB rabbit in all the melanin-containing tissues. However, melanin binding is not a simple process and is influenced by multiple physiological factors. *In vitro* assays demonstrated the melanin binding affinity and capacity of Lev at pH 5 is threefold superior to the parameters measured at pH 7.4 [45]. In addition, the concept of melanosome trapping was recently introduced by Rimpela *et al*. [43, 46]. In summary, the melanosome physiological acidic pH (expected to be between 5 and 6) could change the microstate ionization status of an API within the melanosome thereby rendering the API less permeable upon entering the melanosome, and therefore entrapped within the melanosome. This concept is comparable to lysosomal trapping which is well understood and known to impact API absorption and distribution for oral formulations. Observed data for Lev shows this drug was still detectable in the ICB a month after the single topical administration. The fitted fraction unbound for the three APIs was all 1% or lower. These values could compensate for some physiological mechanisms not implemented in the OCAT model yet. Having access to melanin binding *in vitro* measurements would help to separate the physiological mechanisms affecting fluoroquinolones’ ocular PK. Unfortunately, those data could not be identified in the literature for the test drugs in this research. Nevertheless, the fitted values were aligned with reported *in vitro* measurement for ciprofloxacin, which belongs to the fluoroquinolone drug class too. *In vitro*, ciprofloxacin had the highest binding to melanin among thirty-three APIs tested in cassette assay [47]. Future improvement of the interaction between melanin and APIs in the OCAT model will support its capacity to better predict APIs’ exposure in melanin-containing ocular tissues.

The three APIs used for the validation of the extrapolation strategy proposed in this research are all fluoroquinolone antibiotics. These drugs have the same core structure on which chemical modifications have been made to improve their activity [48]. The clinical PK extrapolation using the OCAT model worked well for these three APIs, but questions remain for other APIs that belong to other chemical families. The API’s physicochemical properties impact its ocular permeabilities [49] and other drug-specific parameters and therefore its ocular penetration and distribution within the ocular tissues. Performing case studies with APIs belonging to multiple chemical families will support the validation effort of the proposed PBPK-based clinical PK extrapolation.

In conclusion, the development, and validation of three PBPK models for APIs administered as ophthalmic solutions were performed based on preclinical data. Those models were successfully applied to predict APIs’ clinical exposure in multiple ocular tissues. This proposed methodology to extrapolate human ocular PK based on a PBPK model validated using preclinical data may support drug development and provide a better understanding of the impact of formulation modifications on the *in vivo* performance of ophthalmic solution products. A deeper understanding of key physiological mechanisms influencing PK outcomes as well as further extrapolation from rabbit to human model for other ophthalmic dosage forms are the next steps planned for this OCAT model.

## Supplementary Information

Below is the link to the electronic supplementary material.

Supplementary file1 (DOCX 1312 KB)

Supplementary file2 (PDF 217 KB)

Supplementary file3 (PDF 581 KB)

## Declarations

### Disclaimer

This article reflects the views of the authors and should not be construed to represent FDA’s views or policies.

## References

[1] Le Merdy M, Fan J, Bolger MB, Lukacova V, Spires J, Tsakalozou E (2019). Application of Mechanistic Ocular Absorption Modeling and Simulation to Understand the Impact of Formulation Properties on Ophthalmic Bioavailability in Rabbits: a Case Study Using Dexamethasone Suspension. AAPS J.

[2] US.FDA. Bioavailability and bioequivalence studies for orally administered drug products — general considerations [Internet]. 2002. Available from: https://www.fda.gov/files/drugs/published/Guidance-for-Industry-Bioavailability-and-Bioequivalence-Studies-for-Orally-Administered-Drug-Products---General-Considerations.PDF. Accessed 1 Sept.

[3] Bellantone RA, Shah KB, Patel PG, Kaplan M, Xu X, Li V (2022). Cyclosporine release and distribution in ophthalmic emulsions determined by pulsatile microdialysis. Int J Pharm.

[4] Choi SH, Lionberger RA (2016). Clinical, pharmacokinetic, and in vitro studies to support bioequivalence of ophthalmic drug products. AAPS J.

[5] Zhao L, Seo P, Lionberger R (2019). Current Scientific Considerations to Verify Physiologically-Based Pharmacokinetic Models and Their Implications for Locally Acting Products. CPT Pharmacomet Syst Pharmacol.

[6] US. FDA. Orange book: approved drug products with therapeutic equivalence evaluations [internet]. 2022. Available from: https://www.accessdata.fda.gov/scripts/cder/ob/. Accessed 1 Sept

[7] Sager JE, Yu J, Ragueneau-Majlessi I, Isoherranen N (2015). Physiologically Based Pharmacokinetic (PBPK) Modeling and Simulation Approaches: A Systematic Review of Published Models, Applications, and Model Verification. Drug Metab Dispos Biol Fate Chem.

[8] Bischoff KB, Dedrick RL, Zaharko DS, Longstreth JA (1971). Methotrexate pharmacokinetics. J Pharm Sci.

[9] Miller NA, Reddy MB, Heikkinen AT, Lukacova V, Parrott N (2019). Physiologically Based Pharmacokinetic Modelling for First-In-Human Predictions: An Updated Model Building Strategy Illustrated with Challenging Industry Case Studies. Clin Pharmacokinet.

[10] Pepin XJH, Parrott N, Dressman J, Delvadia P, Mitra A, Zhang X (2021). Current State and Future Expectations of Translational Modeling Strategies to Support Drug Product Development, Manufacturing Changes and Controls: A Workshop Summary Report. J Pharm Sci.

[11] K Y, Kollipara S, Ahmed T, Chachad S (2022). Applications of PBPK/PBBM modeling in generic product development: An indurstry perspective. J Drug Deliv Sci Technol..

[12] Worakul N, Robinson JR (1997). Ocular pharmacokinetics/pharmacodynamics. Eur J Pharm Biopharm.

[13] US.FDA C for DE and Generic Drug Research Priorities & Projects. FDA [Internet]. 2022 [cited 2022 Apr 26]. Available from: https://www.fda.gov/drugs/generic-drugs/generic-drug-research-priorities-projects. Accessed 1 Sept.

[14] Le Merdy M, Spires J, Lukacova V, Tan ML, Babiskin A, Xu X (2020). Ocular Physiologically Based Pharmacokinetic Modeling for Ointment Formulations. Pharm Res.

[15] Le Merdy M, Tan ML, Babiskin A, Zhao L (2020). Physiologically Based Pharmacokinetic Model to Support Ophthalmic Suspension Product Development. AAPS J.

[16] Koeppe MO, Cristofoletti R, Fernandes EF, Storpirtis S, Junginger HE, Kopp S (2011). Biowaiver monographs for immediate release solid oral dosage forms: levofloxacin. J Pharm Sci.

[17] Stigliani M, Haghi M, Russo P, Young PM, Traini D (2016). Antibiotic transport across bronchial epithelial cells: Effects of molecular weight, LogP and apparent permeability. Eur J Pharm Sci Off J Eur Fed Pharm Sci.

[18] Kłosińska-Szmurło E, Pluciński FA, Grudzień M, Betlejewska-Kielak K, Biernacka J, Mazurek AP (2014). Experimental and theoretical studies on the molecular properties of ciprofloxacin, norfloxacin, pefloxacin, sparfloxacin, and gatifloxacin in determining bioavailability. J Biol Phys.

[19] Destache CJ, Pakiz CB, Larsen C, Owens H, Dash AK (2001). Cerebrospinal fluid penetration and pharmacokinetics of levofloxacin in an experimental rabbit meningitis model. J Antimicrob Chemother.

[20] Dorn C, Nowak H, Weidemann C, Martini S, Zeitlinger M, Adamzik M, et al. Decreased protein binding of moxifloxacin in patients with sepsis? GMS Infect Dis [Internet]. 2017 [cited 2021 Feb 25]; 5. Available from: https://www.ncbi.nlm.nih.gov/pmc/articles/PMC6301732/. Accessed 1 Sept. 10.3205/id000029 PMC6301732 30671325

[21] US.FDA. TEQUIN® FDA Label [Internet]. 2013. Available from: https://www.accessdata.fda.gov/drugsatfda_docs/label/2004/21061s023,024,21062s026,037lbl.pdf. Accessed 1 Sept.

[22] Eedara BB, Tucker IG, Zujovic ZD, Rades T, Price JR, Das SC (2019). Crystalline adduct of moxifloxacin with trans-cinnamic acid to reduce the aqueous solubility and dissolution rate for improved residence time in the lungs. Eur J Pharm Sci Off J Eur Fed Pharm Sci.

[23] Gatifloxacin [Internet]. [cited 2022 Mar 1]. Available from: https://go.drugbank.com/drugs/DB01044. Accessed 1 Sept.

[24] Koeppe MO, Cristofoletti R, Fernandes EF, Storpirtis S, Junginger HE, Kopp S (2011). Biowaiver monographs for immediate release solid oral dosage forms: Levofloxacin. J Pharm Sci.

[25] Lukacova V, Parrott NJ, Fraczkiewicz G, Bolger MB, Woltosz WS. General approach to calculation of tissue: plasma partition coefficients for physiologically based pharmacokinetic (PBPK) modeling. AAPS Annual Meeting; 2008; Atlanta.

[26] Us. FDA. LEVAQUIN® FDA Label [Internet]. 2008. Available from: https://www.fda.gov/files/drugs/published/Levaquin-Label.pdf. Accessed 1 Sept.

[27] US. FDA. AVELOX (moxifloxacin hydrochloride) tablets, for oral use FDA Label [Internet]. 2016. Available from: https://www.accessdata.fda.gov/drugsatfda_docs/label/2016/021085s063lbl.pdf. Accessed 1 Sept.

[28] Chockalingam A, Xu L, Stewart S, LeMerdy M, Tsakalozou E, Fan J (2019). Protocol for evaluation of topical ophthalmic drug products in different compartments of fresh eye tissues in a rabbit model. J Pharmacol Toxicol Methods.

[29] Zaharia AC, Dumitrescu OM, Rogoz RE, Dimirache AE, Zemba M (2021). Preoperative antisepsis in ophthalmic surgery (a review). Romanian J Ophthalmol.

[30] Kumar CM, Seet E, Eke T, Irwin MG, Joshi GP (2019). Peri-operative considerations for sedation-analgesia during cataract surgery: a narrative review. Anaesthesia.

[31] Gower EW, Lindsley K, Nanji AA, Leyngold I, McDonnell PJ (2013). Perioperative antibiotics for prevention of acute endophthalmitis after cataract surgery. Cochrane Database Syst Rev..

[32] George O, Omokhua P (2010). Comparative Analysis Of The Effects Of Topical Anaesthetic Agents On Tear Quantity And Tear Quality. J Niger Optom Assoc.

[33] Jordan A, Baum J (1980). Basic tear flow Does it exist?. Ophthalmology..

[34] Nwaji E, Barrah G (2011). The effect of local anesthetics on tear production. J Niger Optom Assoc.

[35] Cataract | Examination-Based Studies | Information on Data Sources | Vision and Eye Health Surveillance System | Vision Health Initiative (VHI) | CDC [Internet]. 2021 [cited 2022 Mar 27]. Available from: https://www.cdc.gov/visionhealth/vehss/data/studies/cataract.html. Accessed 1 Sept.

[36] Li Y, Fan AZ, Balluz LS (2009). Visual impairment and age-related eye diseases in Florida: Findings from 2006 Behavioral Risk Factors Surveillance System (BRFSS) in Nine states. Risk Manag Healthc Policy.

[37] Karakosta C, Tzamalis A, Aivaliotis M, Tsinopoulos I. Pathogenesis of age-related cataract: a systematic review of proteomic studies. Curr Proteomics. 18(4):458–66.

[38] Nättinen J, Jylhä A, Aapola U, Mäkinen P, Beuerman R, Pietilä J (2019). Age-associated changes in human tear proteome. Clin Proteomics.

[39] Ozdemir M, Temizdemir H (2010). Age- and gender-related tear function changes in normal population. Eye.

[40] Micera A, Di Zazzo A, Esposito G, Longo R, Foulsham W, Sacco R (2018). Age-Related Changes to Human Tear Composition. Invest Ophthalmol Vis Sci.

[41] Singh R, Gupta N, Vanathi M, Tandon R (2019). Corneal transplantation in the modern era. Indian J Med Res.

[42] Al-Yousuf N, Mavrikakis I, Mavrikakis E, Daya SM (2004). Penetrating keratoplasty: indications over a 10 year period. Br J Ophthalmol.

[43] Rimpelä AK, Reinisalo M, Hellinen L, Grazhdankin E, Kidron H, Urtti A (2018). Implications of melanin binding in ocular drug delivery. Adv Drug Deliv Rev.

[44] Durairaj C, Chastain JE, Kompella UB (2012). Intraocular distribution of melanin in human, monkey, rabbit, minipig and dog eyes. Exp Eye Res.

[45] Pelkonen L, Tengvall-Unadike U, Ruponen M, Kidron H, Del Amo EM, Reinisalo M (2017). Melanin binding study of clinical drugs with cassette dosing and rapid equilibrium dialysis inserts. Eur J Pharm Sci Off J Eur Fed Pharm Sci.

[46] Bahrpeyma S, Rimpelä AK, Hagström M, Urtti A. Ocular melanin binding of drugs: in vitro binding studies. Acta Ophthalmol (Copenh) [Internet]. 2019 [cited 2022 Mar 28];97(S263). Available from: 10.1111/j.1755-3768.2019.5366

[47] Rimpelä AK, Hagström M, Kidron H, Urtti A (2018). Melanin targeting for intracellular drug delivery: Quantification of bound and free drug in retinal pigment epithelial cells. J Control Release Off J Control Release Soc.

[48] Pham TDM, Ziora ZM, Blaskovich MAT (2019). Quinolone antibiotics. MedChemComm.

[49] Prausnitz MR, Noonan JS (1998). Permeability of cornea, sclera, and conjunctiva: a literature analysis for drug delivery to the eye. J Pharm Sci.

