# Supplementary material for: Clinical Ocular Exposure Extrapolation for Ophthalmic Solutions Using PBPK Modeling and Simulation
Source: Pharm Res. 2022 Sep 23;40(2):431–47. doi: 10.1007/s11095-022-03390-z (PMC9944674; doi:10.1007/s11095-022-03390-z)
Supplement: Supplementary file 1 — Supplementary file1 (DOCX 1312 KB) [file 11095_2022_3390_MOESM1_ESM.docx]

### Clinical Ocular Exposure Extrapolation for Ophthalmic Solutions Using PBPK Modeling and Simulation

Maxime Le Merdy^1^, Farah AlQaraghuli^1^, Ming-Liang Tan^2^, Ross Walenga^2^, Andrew Babiskin^2^, Liang Zhao^2^, Viera Lukacova^1^

*1: Simulations Plus, Inc., 42505 10th Street West, Lancaster, California 93534, USA.*

*2: Division of Quantitative Methods and Modeling, Office of Research and Standards, Office of Generic Drugs, Center for Drug Evaluation and Research, U.S. Food and Drug Administration, 10903 New Hampshire Avenue, Silver Spring, MD 20993, USA*

**Supplementary material 1:**

**Systemic PBPK Model**

[Table 1: List of Data used to develop the Lev rabbit and Human PBPK models. 2](#_Toc98867316)

[Table 2: List of Data used to develop the Mox rabbit and Human PBPK models. 4](#_Toc98867317)

[Table 3: List of Data used to develop the Gat rabbit and Human PBPK models. 7](#_Toc98867318)

[Figure 1: Observed (squares) and simulated (lines) Levofloxacin Plasma Concentration and Urine Time Courses Following IV Bolus Administration of 7, 10.5, and 14 mg/kg to Rabbits (1). 2](#_Toc98867319)

[Figure 2: Observed (squares) and Simulated (lines) Levofloxacin Plasma Concentration Time Course Following multiple IV (left) and PO (right) Administrations to Healthy Male Subjects (2). 3](#_Toc98867320)

[Figure 3: Observed (squares) and Simulated (lines) Moxifloxacin Plasma Concentration and Urine Time Course Following IV Bolus (left) and PO (right) Administrations of 17.5 mg (5 mg/kg) to Rabbits. 5](#_Toc98867321)

[Figure 4: Observed (squares) and Simulated (lines) Moxifloxacin Plasma Concentration and Urine Time Course Following IV and PO Administration of 100 mg to Healthy Human Male Subjects. 6](#_Toc98867322)

[Figure 5: Observed (squares) and Simulated (lines) Gatifloxacin Plasma Concentration and Urine Time Course Following IV (Top row) and PO (bottom row) Administration of 400 mg to Healthy Human Male (left panels) and Female (right panels) Subjects. 8](#_Toc98867323)

**Levofloxacin**

*Data*

Data used to develop rabbit and human levofloxacin (Lev) PBPK model are listed in Table 1:

**Table 1: List of Data used to develop the Lev rabbit and Human PBPK models.**

**ROA: route of administration; IV: intravenous administration; PO: oral administration**

| Species | ROA | Dose | Source |
| --- | --- | --- | --- |
| Rabbit | IV | 7 - 10.5 - 14 mg/kg | (1) |
| Human | IV | 500 mg | (2) |
| Human | PO | 500 mg | (2) |

*Rabbit model*

Plasma concentration time course data following IV bolus administration of Lev (7, 10.5, and 14 mg/kg) to rabbits (1) were used to describe the systemic model. A non-compartmental analysis (NCA) of the observed data estimated the CL and Vd to be 0.537 L/h (0.215 L/h/kg) and 3.741 L (1.496 L/kg), respectively. The baseline PBPK model in rabbits was developed with all tissues defined as perfusion limited. The Kps values were calculated using the default Lukacova method. Blood/Plasma concentration ratio (Rbp) was adjusted to 0.95 to predict the Vd based on the NCA results. The final predicted Vd was 4.696 L. Renal clearance was set to equal the fraction of kidney blood flow and the fraction was fitted to 0.1 to capture the total amount of drug eliminated in urine (87%) (3). A liver clearance parameter was fitted so the addition of renal and liver clearances would be comparable to the NCA calculated one. An intrinsic liver clearance of 0.054 L/h was used for rabbit simulations. The final model prediction for the 7, 10.5, and 14 mg/kg dose is presented in Figure 1. All the observed data could be nicely overlaid with the simulations, thereby validating the baseline PBPK model in Rabbit.


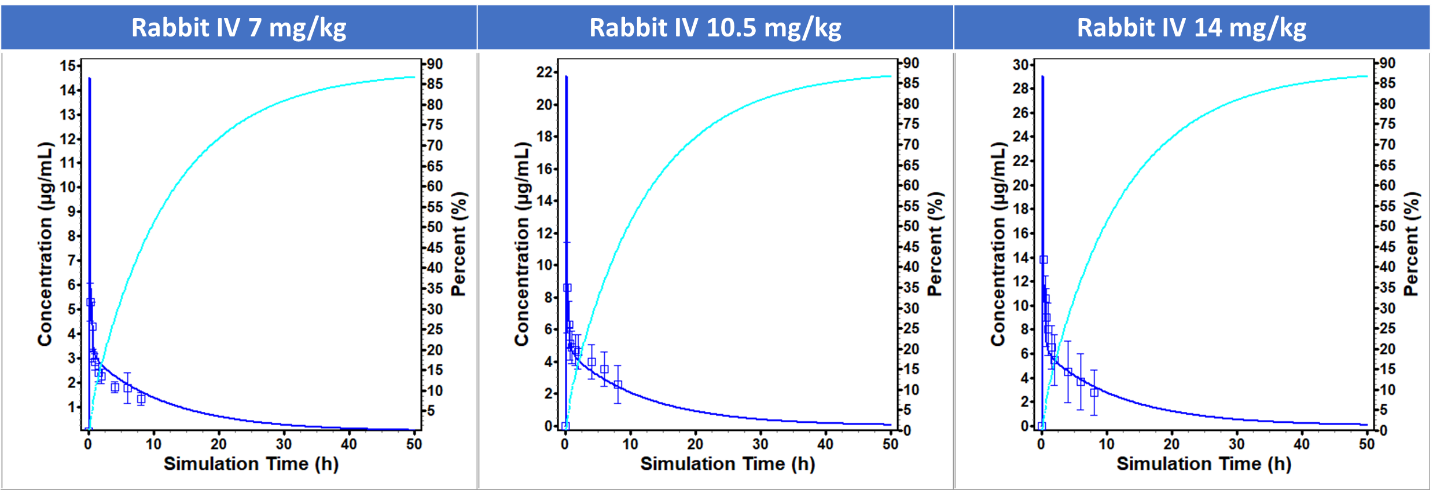


**Figure 1: Observed (squares) and simulated (lines) Levofloxacin Plasma Concentration and Urine Time Courses Following IV Bolus Administration of 7, 10.5, and 14 mg/kg to Rabbits (1).**

**Dark blue lines represent plasma concentrations (left Y axis) and cyan lines represent percent of dose secreted in urine (right Y axis)**

*Human model*

Plasma concentration time course data following IV bolus administration of Lev (500 mg) to healthy male subjects (2) was used to describe the systemic distribution and elimination model. A NCA of the observed data estimated the clearance and Vd to be 8.923 L/h (0.095 L/h/kg) and 79.46 L (0.842 L/kg), respectively. The human baseline PBPK model was developed with all tissues defined as perfusion limited. The Kps values were calculated using the default Lukacova method. Similar to rabbit model, the Rbp was adjusted to capture the NCA calculated Vd. The fitted human Rbp value was 0.84 and the final predicted Vd was 79.475 L. Renal clearance was again set to equal the fraction of kidney blood flow and the fraction was fitted to 0.09 to capture the total amount of drugs eliminated in urine (87%) (3). Similar to the rabbit PBPK model, a liver clearance parameter was fitted so the addition of renal and liver clearances would be comparable to the NCA calculated one. An intrinsic liver clearance of 2 L/h was used for human simulations. The final model prediction is presented in Figure 2. Chien et al. also reported concentration time course following the oral administration of 488 mg (2). In vivo gastrointestinal permeability was scaled form in vitro data (4). Without making any adjustment to the model, those data were simulated. Prediction of Lev plasma concentration time course following oral administration are presented in Figure 2. Overall, the PBPK model can predict Lev pharmacokinetic following both IV and oral administration in human.


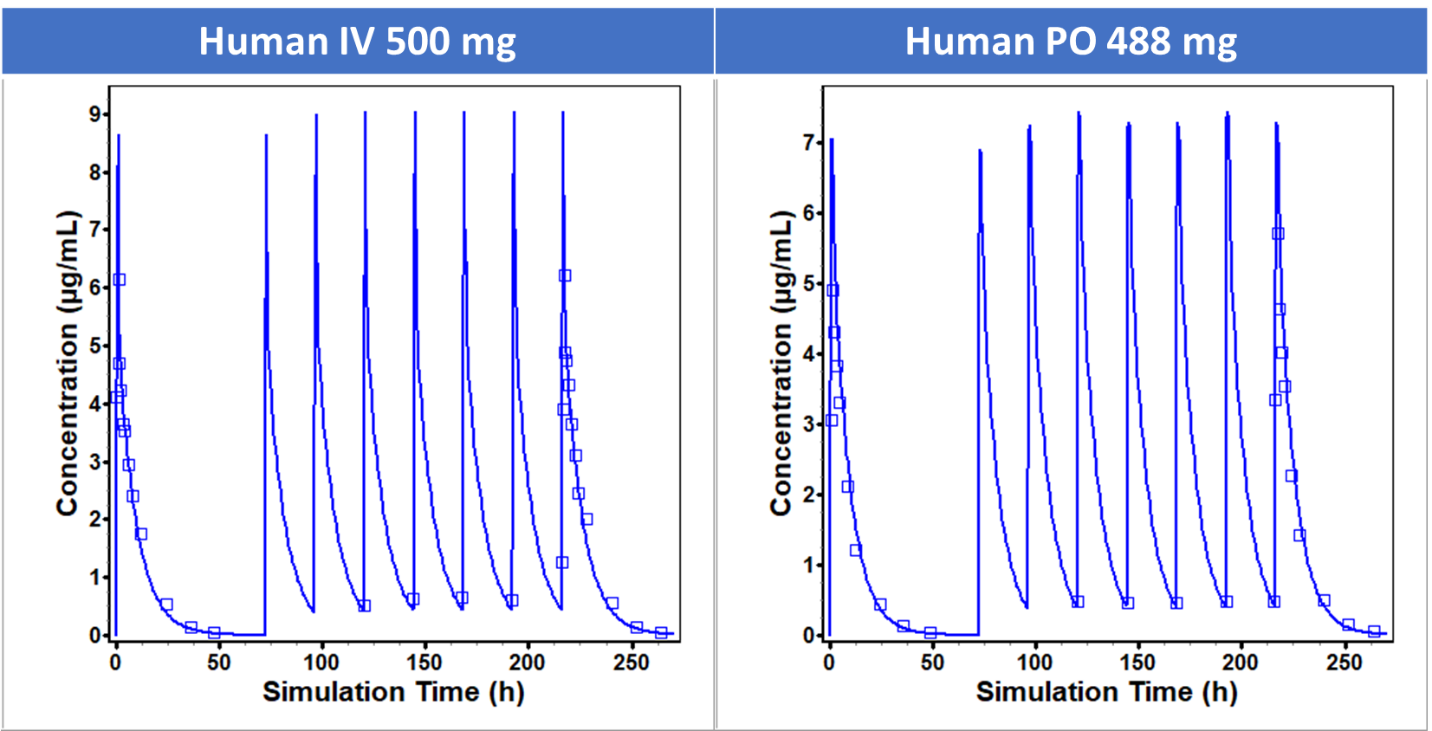


**Figure 2: Observed (squares) and Simulated (lines) Levofloxacin Plasma Concentration Time Course Following multiple IV (left) and PO (right) Administrations to Healthy Male Subjects (2).**

**Moxifloxacin**

*Data*

Data used to develop rabbit and human moxifloxacin (Mox) PBPK model are listed in Table 2:

**Table 2: List of Data used to develop the Mox rabbit and Human PBPK models.**

**ROA: route of administration; IV: intravenous administration; PO: oral administration**

| Species | ROA | Dose | Source |
| --- | --- | --- | --- |
| Rabbit | IV | 5 mg/kg | (5) |
| Rabbit | PO | 5 mg/kg | (5) |
| Human | IV | 100 mg | (6) |
| Human | PO | 100 mg | (6) |

*Rabbit model*

Plasma concentration time course data following IV bolus and PO administration of Mox (5 mg/kg) to rabbits (5) were used to describe the systemic model. A NCA of the observed data following the IV administration estimated the CL and Vd to be 2.789 L/h (0.797 L/h/kg) and 6.454 L (1.844 L/kg), respectively. The baseline PBPK model in rabbits was developed with all tissues defined as perfusion limited. The Kps values were calculated using the default Lukacova method. Rbp was adjusted to 0.9 to predict the Vd based on the NCA results. The final predicted Vd was 4.696 L. Renal clearance was set to equal the fraction of kidney blood flow and the fraction was fitted to 0.11 to match total renal excretion of 20% (7). Liver clearance was then fit to capture the observed data (Liver Clint = 3.7 L/h). The final model prediction for the 17.5 mg (5 mg/kg) IV dose is presented in Figure 3 (left). This developed model was then tested to simulate the PO solution administration. The observed PO data were nicely overlaid by the simulations once the intestinal Peff was adjusted to 0.1*10^-4^ cm/s and the gastric transit time changed to 0.25 hr, which was likely due to known faster transit times of solution formulations (Figure 3, right). The baseline PBPK model in rabbit was then considered validated.


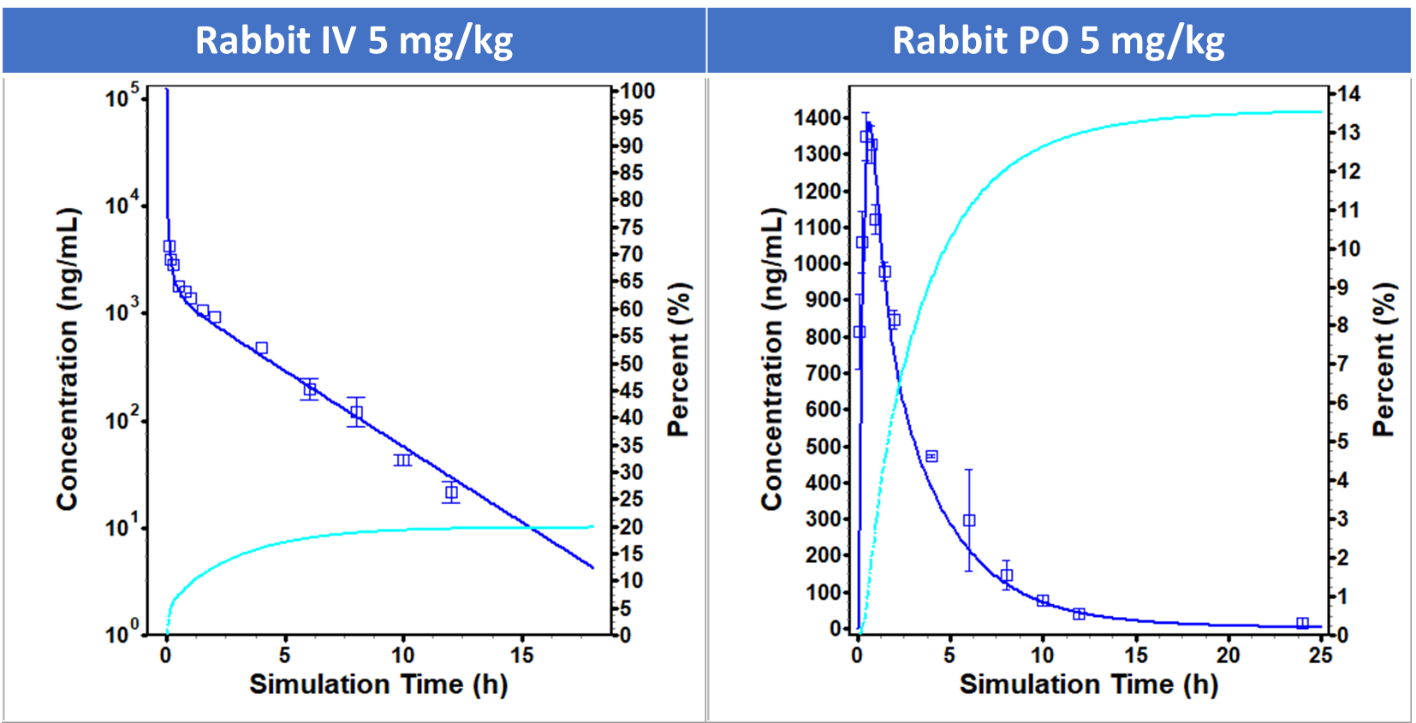


**Figure 3: Observed (squares) and Simulated (lines) Moxifloxacin Plasma Concentration and Urine Time Course Following IV Bolus (left) and PO (right) Administrations of 17.5 mg (5 mg/kg) to Rabbits.**

**Dark blue lines represent plasma concentrations (left Y axis) and cyan lines represent percent of dose secreted in urine (right Y axis)**

*Human model*

Plasma concentration time course data following IV and PO administration of Mox (100 mg) to healthy male subjects (6) were used to describe the systemic distribution and elimination model. An NCA of the observed data estimated the clearance and Vd to be 7.929 L/h (0.102 L/h/kg) and 140.9 L (1.814 L/kg), respectively. The human baseline PBPK model was developed with all tissues defined as perfusion limited. The Kps were calculated using the default Lukacova method. Initial Vd estimation was done with the same optimized Rbp parameter (0.9) used in the rabbit simulation. This led to a good estimation of the Vd, with a final predicted Vd of 135.1 L. Renal clearance was again set to equal the fraction of kidney blood flow, but the fraction had to be specifically fit to match 20% urinary excretion (final fraction of 0.02) (7). Liver clearance was then fit to describe the observed data for the IV administration (Liver Clint = 8 L/h). Simulation results are presented in Figure 4 (left). For the PO administration, Peff needed to be adjusted to 4*10^-4^ cm/s to describe the observed data (Figure 4, right).


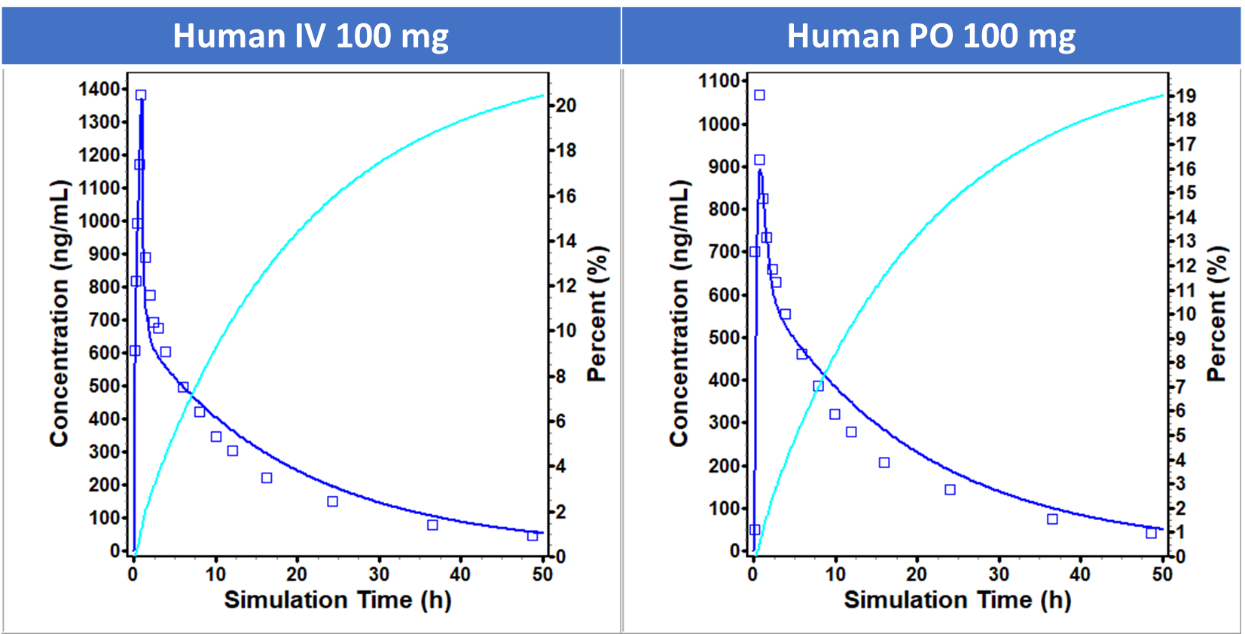


**Figure 4: Observed (squares) and Simulated (lines) Moxifloxacin Plasma Concentration and Urine Time Course Following IV and PO Administration of 100 mg to Healthy Human Male Subjects.**

**Dark blue lines represent plasma concentrations (left Y axis) and cyan lines represent percent of dose secreted in urine (right Y axis)**

**Gatifloxacin**

*Data*

Data used to develop rabbit and human gatifloxacin (Gat) PBPK model are listed in Table 3:

**Table 3: List of Data used to develop the Gat rabbit and Human PBPK models.**

**ROA: route of administration; IV: intravenous administration; PO: oral administration**

| Species | ROA | Dose | Source |
| --- | --- | --- | --- |
| Human | IV | 400 | (8) |
| Human | PO | 400 | (8) |

*Rabbit model*

No IV or oral data following administration to rabbit were identified in the public domain. Therefore, the rabbit PBPK model was adjusted based on the human PBPK development (see next section for details). In summary, Rbp was adjusted to 0.9 to predict the Vd based. Renal clearance was set to equal the fraction of kidney blood flow and this fraction was fit to 0.12 to match human parameter. The ratios of human renal and liver clearances were used to estimate rabbit liver clearance. For rabbit, the liver Clint was set to 0.16 L/h.

*Human model*

Plasma concentration time course data following IV and PO administration of Gat (400 mg) to healthy male and female subjects (8) were used to describe the systemic distribution and elimination model. An NCA of the observed male data estimated the clearance and Vd to be 11.28 L/h (0.150 L/h/kg) and 101 L (1.347 L/kg), respectively. The human baseline PBPK model was developed with all tissues defined as perfusion limited. The Kps were calculated using the default Lukacova method. Rbp was adjusted to 0.9 to predict the Vd based on the NCA results. The final predicted Vd was 96.4 L. Renal clearance was set to equal the fraction of kidney blood flow and the fraction was fitted to 0.12 to match total renal excretion of 80% (9). Liver clearance was then fit to capture the observed data (Liver Clint = 2.7 L/h). The final model prediction for the 400 mg IV dose in healthy male is presented in Figure 5 (top left). This developed model was then tested to simulate the IV administration in female. The observed IV data was nicely overlaid by the simulations (Figure 5, top right). The baseline PBPK model in human was then considered validated. To capture the oral data in both male and female, Peff needed to be adjusted to 2*10^-4^ cm/s to describe the observed data (Figure 5, bottom row).


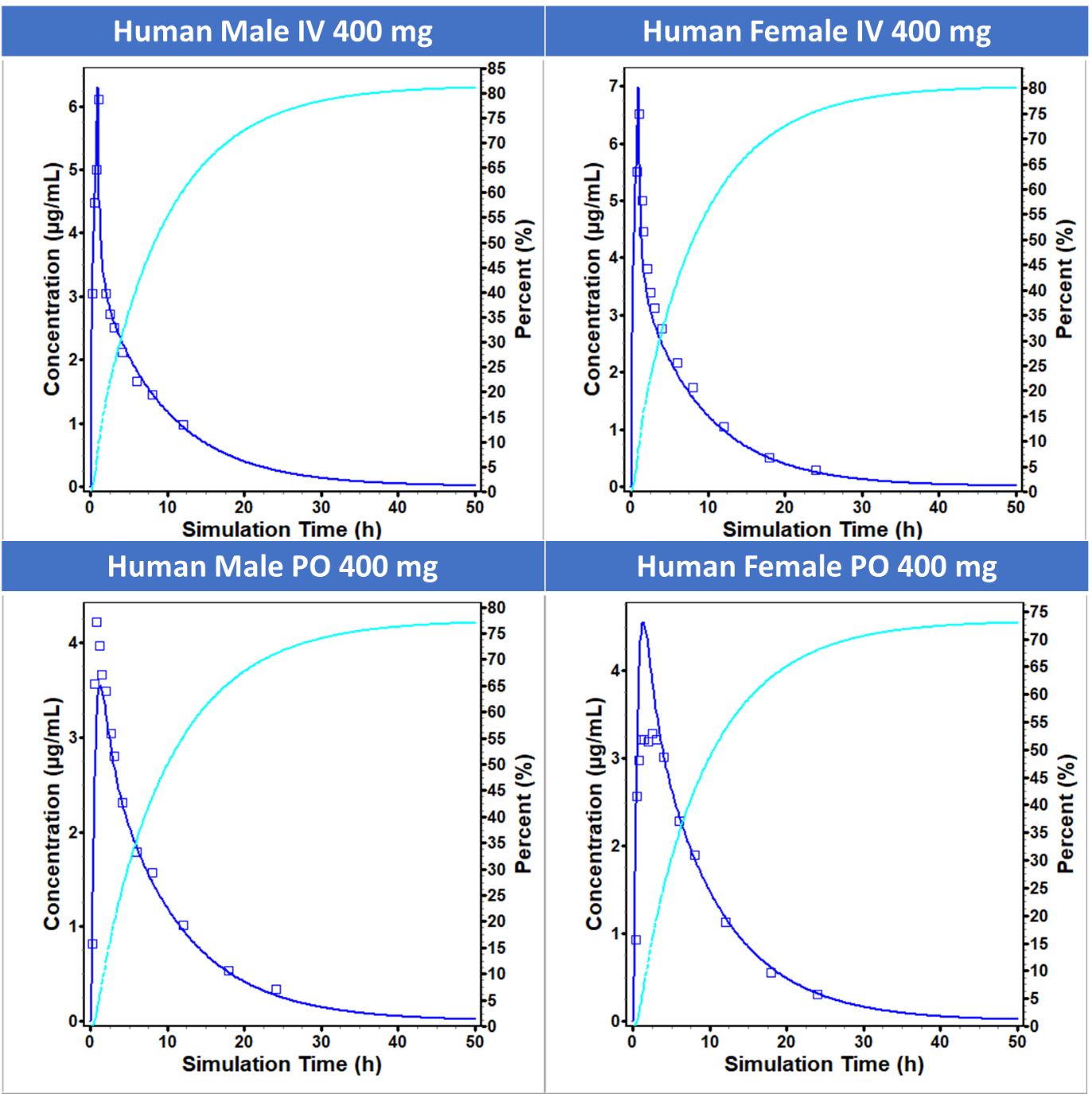


**Figure 5: Observed (squares) and Simulated (lines) Gatifloxacin Plasma Concentration and Urine Time Course Following IV (Top row) and PO (bottom row) Administration of 400 mg to Healthy Human Male (left panels) and Female (right panels) Subjects.**

**Dark blue lines represent plasma concentrations (left Y axis) and cyan lines represent percent of dose secreted in urine (right Y axis)**

**References**

1. Destache CJ, Pakiz CB, Larsen C, Owens H, Dash AK. Cerebrospinal fluid penetration and pharmacokinetics of levofloxacin in an experimental rabbit meningitis model. J Antimicrob Chemother. 2001 May 1;47(5):611–5.

2. Chien SC, Rogge MC, Gisclon LG, Curtin C, Wong F, Natarajan J, et al. Pharmacokinetic profile of levofloxacin following once-daily 500-milligram oral or intravenous doses. Antimicrob Agents Chemother. 1997 Oct;41(10):2256–60.

3. Us. FDA. LEVAQUIN® FDA Label [Internet]. 2008. Available from: https://www.fda.gov/files/drugs/published/Levaquin-Label.pdf

4. Koeppe MO, Cristofoletti R, Fernandes EF, Storpirtis S, Junginger HE, Kopp S, et al. Biowaiver monographs for immediate release solid oral dosage forms: Levofloxacin. J Pharm Sci. 2011;100(5):1628–36.

5. Fernández-Varón E, Bovaira MJ, Espuny A, Escudero E, Vancraeynest D, Cárceles CM. Pharmacokinetic-pharmacodynamic integration of moxifloxacin in rabbits after intravenous, intramuscular and oral administration. J Vet Pharmacol Ther. 2005 Aug;28(4):343–8.

6. Ballow C, Lettieri J, Agarwal V, Liu P, Stass H, Sullivan JT. Absolute bioavailability of moxifloxacin. Clin Ther. 1999 Mar 1;21(3):513–22.

7. US. FDA. AVELOX (moxifloxacin hydrochloride) tablets, for oral use FDA Label [Internet]. 2016. Available from: https://www.accessdata.fda.gov/drugsatfda_docs/label/2016/021085s063lbl.pdf

8. LaCreta FP, Kaul S, Kollia GD, Duncan G, Randall DM, Grasela DM. Interchangeability of 400-mg intravenous and oral gatifloxacin in healthy adults. Pharmacotherapy. 2000 Jun;20(6 Pt 2):59S-66S.

9. US.FDA. TEQUIN® FDA Label [Internet]. 2013. Available from: https://www.accessdata.fda.gov/drugsatfda_docs/label/2004/21061s023,024,21062s026,037lbl.pdf
