# Supplementary material for: Clinical Ocular Exposure Extrapolation for Ophthalmic Solutions Using PBPK Modeling and Simulation
Source: Pharm Res. 2022 Sep 23;40(2):431–47. doi: 10.1007/s11095-022-03390-z (PMC9944674; doi:10.1007/s11095-022-03390-z)
Supplement: Supplementary file 2 — Supplementary file2 (PDF 217 KB) [file 11095_2022_3390_MOESM2_ESM.pdf]

Clinical Ocular Exposure Extrapolation for Ophthalmic Solutions Using PBPK Modeling and Simulation

Maxime Le Merdy[1], Farah AlQaraghuli[1], Ming-Liang Tan[2], Ross Walenga[2], Andrew Babiskin[2], Liang Zhao[2], Viera Lukacova[1]

1: Simulations Plus, Inc., 42505 10th Street West, Lancaster, California 93534, USA.
2: Division of Quantitative Methods and Modeling, Office of Research and Standards, Office of Generic Drugs, Center for Drug Evaluation and Research, U.S. Food and Drug Administration, 10903 New Hampshire Avenue, Silver Spring, MD 20993, USA

Supplementary material 2: Ophthalmic Preclinical-Clinical data

Table 1: List of preclinical studies with available ocular tissues concentrations used for Levofloxacin OCAT model development and validation 2
Table 4: List of clinical studies with available ocular tissues concentrations used for Human extrapolation using the Levofloxacin OCAT model 2
Table 2: List of preclinical studies with available ocular tissues concentrations used for Moxifloxacin OCAT model development and validation 3
Table 5: List of clinical studies with available ocular tissues concentrations used for Human extrapolation using the Moxifloxacin OCAT model 3
Table 3: List of preclinical studies with available ocular tissues concentrations used for Gatifloxacin OCAT model development and validation 4
Table 6: List of clinical studies with available ocular tissues concentrations used for Human extrapolation using the Gatifloxacin OCAT model 5

Levofloxacin

Table 1: List of preclinical studies with available ocular tissues concentrations used for Levofloxacin OCAT model development and validation

Study code	Species	Conc (%W/V)	Dose	Volume (µL)	Tissue of Interest	Source
Lev.NZ.1	JW	1.5	single	30	Cornea, Conj	(1)
Lev.NZ.2	NZ	1.5	single	50	Cornea, AH	(2)
Lev.NZ.3	NZ	1.5	single	50	Cornea, AH	(3)
Lev.NZ.4	NZ	0.5	multiple	50	Cornea, AH, ICB, VH	(4)
Lev.NZ.5	NZ	0.5	multiple	50	AH, VH	(5)
Lev.NZ.6	NZ	0.5	multiple	50	AH	(6)
Lev.NZ.7	NZ	0.5	multiple	50	Cornea, Conj, AH	(7)
Lev.DB.1	DB	0.5	single	50	Cornea, AH, ICB, VH, Sclera	(8)

NZ = New Zealand Rabbit; DB = Dutch Belted Rabbit; JW = Japanese white Rabbit (assumed similar to NZ); Conj = Conjunctiva; AH= Aqueous Humor; VH = Vitreous Humor; ICB = Iris Ciliary Body: Conc: Solution Concentration

Table 2: List of clinical studies with available ocular tissues concentrations used for Human extrapolation using the Levofloxacin OCAT model

Study Code	Surgery	Conc (%W/V)	Dose	Volume (µL)	Tissue of Interest	Source
Lev.Hum.1	cataract	0.5%	multiple	39	AH	(9)
Lev.Hum.2	cataract	0.5%	multiple	39	AH	(10)
Lev.Hum.3	cataract	0.5%	multiple	39	AH	(11)
Lev.Hum.4	cataract	1.50%	multiple	39	AH	(12)
Lev.Hum.5	keratoplasty	1.5%	multiple	39	Cornea, AH	(13)
Lev.Hum.6	keratoplasty	0.5%	multiple	39	Cornea, AH	(14)
Lev.Hum.7	keratoplasty	0.5%	multiple	39	Cornea	(15)
Lev.Hum.8	vitrectomy	0.5%	multiple	39	AH, VH	(16)
Lev.Hum.9	vitrectomy	0.5%	multiple	39	VH	(17)

AH= Aqueous Humor; VH = Vitreous Humor: Conc: Solution Concentration

Moxifloxacin

Table 3: List of preclinical studies with available ocular tissues concentrations used for Moxifloxacin OCAT model development and validation

Study code	strain	Conc (%W/V)	Dose	Volume (µL)	Tissue of Interest	Source
Mox.NZ.1	NZ	0.5	single	50	Cornea, AH	(2)
Mox.NZ.2	NZ	0.5	single	30	AH	(18)
Mox.NZ.3	NZ	0.5	multiple	50	AH	(19)
Mox.NZ.4	NZ	0.5	multiple	50	AH	(19)
Mox.NZ.5	NZ	0.5	multiple	50	Cornea, ICB, AH, VH	(4)
Mox.DB.1	DB	0.3	single	30	Cornea, AH, ICB	(20)
Mox.DB.2	DB	0.5	single	50	Cornea, Conj, AH, VH	(21)

NZ = New Zealand Rabbit; DB = Dutch Belted Rabbit; Conj = Conjunctiva; AH= Aqueous Humor; VH = Vitreous Humor; ICB = Iris Ciliary Body: Conc: Solution Concentration

Table 4: List of clinical studies with available ocular tissues concentrations used for Human extrapolation using the Moxifloxacin OCAT model

Study Code	Surgery	Conc (%W/V)	Dose	Volume (µL)	Tissue of Interest	Source
Mox.Hum.1	cataract	0.5	single	39	AH	(22)
Mox.Hum.2	cataract	0.5	multiple	39	AH	(23)
Mox.Hum.3	cataract	0.5	multiple	39	AH	(23)
Mox.Hum.4	cataract	0.5	multiple	39	AH	(24)
Mox.Hum.5	cataract	0.5	multiple	39	AH	(25)
Mox.Hum.6	cataract	0.5	multiple	39	AH	(25)
Mox.Hum.7	cataract	0.5	multiple	39	AH	(26)
Mox.Hum.8	cataract	0.5	multiple	39	AH	(27)
Mox.Hum.9	cataract	0.5	multiple	39	AH	(27)
Mox.Hum.10	cataract	0.5	multiple	39	AH	(28)
Mox.Hum.11	cataract	0.5	multiple	39	AH	(12)
Mox.Hum.12	keratoplasty	0.3	multiple	39	Cornea, AH	(29)
Mox.Hum.13	keratoplasty	0.5	multiple	39	Cornea, AH	(14)
Mox.Hum.14	vitrectomy	0.5	multiple	39	AH, VH	(30)
Mox.Hum.15	vitrectomy	0.5	multiple	39	AH, VH	(30)
Mox.Hum.16	vitrectomy	0.5	multiple	39	VH	(31)
Mox.Hum.17	vitrectomy	0.5	multiple	39	VH	(31)
Mox.Hum.18	healthy	0.5	single	39	Conjunctival	(32)
Mox.Hum.19	healthy	0.5	single	39	Conjunctival	(33)

AH= Aqueous Humor; VH = Vitreous Humor: Conc: Solution Concentration

Gatifloxacin

Table 5: List of preclinical studies with available ocular tissues concentrations used for Gatifloxacin OCAT model development and validation

Study code	Specie	Conc (%W/V)	Dose	Volume (μL)	Tissue of Interest	Source
Gat.NZ.1	NZ	0.3	single	50	Cornea, AH	(2)
Gat.NZ.2	NZ	0.3	single	50	AH	(34)
Gat.NZ.3	NZ	0.3	single	50	AH	(35)
Gat.NZ.4	NZ	0.3	single	40	AH	(36)
Gat.NZ.5	NZ	0.3	single	50	AH	(37)
Gat.NZ.6	JW	0.3	multiple	50	AH	(38)
Gat.NZ.7	NZ	0.3	multiple	50	Cornea, ICB, AH, VH	(4)
Gat.NZ.8	NZ	0.3	multiple	50	AH	(6)
Gat.NZ.9	NZ	0.3	multiple	50	AH	(19)
Gat.NZ.10	NZ	0.3	multiple	50	AH	(19)
Gat.DB.1	DB	0.3	single	50	Conj Tears	(21)
Gat.DB.2	DB	0.3	multiple	50	Cornea, ICB, AH, VH	(20)

NZ = New Zealand Rabbit; DB = Dutch Belted Rabbit; JW = Japanese white Rabbit (assumed similar to NZ); Conj = Conjunctiva; AH= Aqueous Humor; VH = Vitreous Humor; ICB = Iris Ciliary Body: Conc: Solution Concentration

Table 6: List of clinical studies with available ocular tissues concentrations used for Human extrapolation using the Gatifloxacin OCAT model

Study Code	Surgery	Conc (%W/V)	Dose	Volume (µL)	Tissue of Interest	Source
Gat.Hum.1	cataract	0.3	single	39	AH	(22)
Gat.Hum.2	cataract	0.3	multiple	39	AH	(24)
Gat.Hum.3	cataract	0.3	multiple	39	AH	(27)
Gat.Hum.4	cataract	0.3	multiple	39	AH	(27)
Gat.Hum.5	cataract	0.3	multiple	39	AH	(25)
Gat.Hum.6	cataract	0.3	multiple	39	AH	(25)
Gat.Hum.7	cataract	0.3	multiple	39	AH	(26)
Gat.Hum.8	cataract	0.3	multiple	39	AH	(39)
Gat.Hum.9	cataract	0.3	multiple	39	AH	(40)
Gat.Hum.10	cataract	0.3	multiple	39	AH	(41)
Gat.Hum.11	cataract	0.3	multiple	39	AH	(9)
Gat.Hum.12	cataract	0.3	multiple	39	AH	(28)
Gat.Hum.13	keratoplasty	0.3	multiple	39	Cornea, AH	(13)
Gat.Hum.14	keratoplasty	0.3	multiple	39	Cornea, AH	(29)
Gat.Hum.15	keratoplasty	0.3	multiple	39	Cornea, AH	(14)
Gat.Hum.16	vitrectomy	0.3	multiple	39	VH	(31)
Gat.Hum.17	vitrectomy	0.3	multiple	39	VH	(31)
Gat.Hum.18	healthy	0.3	single	39	Conjunctival	(32)
Gat.Hum.19	healthy	0.3	single	39	Conjunctival	(33)

AH= Aqueous Humor; VH = Vitreous Humor: Conc: Solution Concentration

References

1. Sakai T, Shinno K, Kurata M, Kawamura A. Pharmacokinetics of Azithromycin, Levofloxacin, and Ofloxacin in Rabbit Extraocular Tissues After Ophthalmic Administration. Ophthalmol Ther. 2019 Dec;8(4):511–7.
2. Chung JL, Lim EH, Song SW, Kim BY, Lee JH, Mah FS, et al. Comparative intraocular penetration of 4 fluoroquinolones after topical instillation. Cornea. 2013 Jul;32(7):1046–51.
3. Bezwada P, Clark L, Adams S, O'Brien T, Schultz G. Comparative Ocular Bioavailability and Efficacy of Topical Levofloxacin and Ofloxacin in Rabbits. J Toxicol Cutan Ocul Toxicol. 2005 Jan 1;23(2):83–90.
4. Fukuda M, Sasaki H. Calculation of AQCmax: comparison of five ophthalmic fluoroquinolone solutions*. Curr Med Res Opin. 2008 Dec;24(12):3479–86.
5. Yağci R, Oflu Y, Dinçel A, Kaya E, Yağci S, Bayar B, et al. Penetration of second-, third-, and fourth-generation topical fluoroquinolone into aqueous and vitreous humour in a rabbit endophthalmitis model. Eye Lond Engl. 2007 Jul;21(7):990–4.
6. Levine JM, Noecker RJ, Lane LC, Snyder RW, Rapedius M, Blanchard J. Aqueous penetration of gatifloxacin and levofloxacin into the rabbit aqueous humor following topical dosing. J Ocul Pharmacol Ther Off J Assoc Ocul Pharmacol Ther. 2004 Jun;20(3):210–6.
7. Li G, Xu L, Jiang M, Wu X. Eye drops and eye gels of levofloxacin: comparison of ocular absorption characterizations and therapeutic effects in the treatment of bacterial keratitis in rabbits. Drug Dev Ind Pharm. 2020 Apr;46(4):673–81.
8. Ibuki H. OkaK. Ocular Pharmacokinetics of Levofloxacin (LVFX) after instillation to pigmented Rabbit Eyes. Atarashii Ganka. 1997;14 (7):1100–4.
9. Ding W, Ni W, Chen H, Yuan J, Huang X, Zhang Z, et al. Comparison of Drug Concentrations in Human Aqueous Humor after the Administration of 0.3% Gatifloxacin Ophthalmic Gel, 0.3% Gatifloxacin and 0.5% Levofloxacin Ophthalmic Solutions. Int J Med Sci. 2015 Jun 10;12(6):517–23.
10. Koch H-R, Kulus SC, Roessler M, Ropo A, Geldsetzer K. Corneal penetration of fluoroquinolones: aqueous humor concentrations after topical application of levofloxacin 0.5% and ofloxacin 0.3% eyedrops. J Cataract Refract Surg. 2005 Jul;31(7):1377–85.
11. Yamada M, Mochizuki H, Yamada K, Kawai M, Mashima Y. Aqueous humor levels of topically applied levofloxacin in human eyes. Curr Eye Res. 2002 May;24(5):403–6.
12. Bucci FA, Nguimfack IT, Fluet AT. Pharmacokinetics and aqueous humor penetration of levofloxacin 1.5% and moxifloxacin 0.5% in patients undergoing cataract surgery. Clin Ophthalmol Auckl NZ. 2016;10:783–9.
13. Holland EJ, McCarthy M, Holland S. The ocular penetration of levofloxacin 1.5% and gatifloxacin 0.3% ophthalmic solutions in subjects undergoing corneal transplant surgery. Curr Med Res Opin. 2007 Jan 1;23(12):2955–60.
14. Fukuda M, Yamada M, Kinoshita S, Inatomi T, Ohashi Y, Uno T, et al. Comparison of corneal and aqueous humor penetration of moxifloxacin, gatifloxacin and levofloxacin during keratoplasty. Adv Ther. 2012 Apr;29(4):339–49.
15. Yamada M, Ishikawa K, Mochizuki H, Kawai M. Corneal penetration of simultaneously applied topical levofloxacin, norfloxacin and lomefloxacin in human eyes. Acta Ophthalmol Scand. 2006 Apr;84(2):192–6.

16. Sakamoto H, Sakamoto M, Hata Y, Kubota T, Ishibashi T. Aqueous and vitreous penetration of levofloxacin after topical and/or oral administration. Eur J Ophthalmol. 2007 Jun;17(3):372–6.
17. Puustjärvi T, Teräsvirta M, Nurmenniemi P, Lokkila J, Uusitalo H. Penetration of topically applied levofloxacin 0.5% and ofloxacin 0.3% into the vitreous of the non-inflamed human eye. Graefes Arch Clin Exp Ophthalmol Albrecht Von Graefes Arch Klin Exp Ophthalmol. 2006 Dec;244(12):1633–7.
18. Jain GK, Jain N, Pathan SA, Akhter S, Talegaonkar S, Chander P, et al. Ultra high-pressure liquid chromatographic assay of moxifloxacin in rabbit aqueous humor after topical instillation of moxifloxacin nanoparticles. J Pharm Biomed Anal. 2010 May 1;52(1):110–3.
19. Levine JM, Noecker RJ, Lane LC, Herrygers L, Nix D, Snyder RW. Comparative penetration of moxifloxacin and gatifloxacin in rabbit aqueous humor after topical dosing. J Cataract Refract Surg. 2004 Oct;30(10):2177–82.
20. Robertson SM, Curtis MA, Schlech BA, Rusinko A, Owen GR, Dembinska O, et al. Ocular pharmacokinetics of moxifloxacin after topical treatment of animals and humans. Surv Ophthalmol. 2005 Nov;50 Suppl 1:S32-45.
21. Proksch JW, Ward KW. Ocular pharmacokinetics/pharmacodynamics of besifloxacin, moxifloxacin, and gatifloxacin following topical administration to pigmented rabbits. J Ocul Pharmacol Ther Off J Assoc Ocul Pharmacol Ther. 2010 Oct;26(5):449–58.
22. Donnenfeld ED, Comstock TL, Proksch JW. Human aqueous humor concentrations of besifloxacin, moxifloxacin, and gatifloxacin after topical ocular application. J Cataract Refract Surg. 2011 Jun;37(6):1082–9.
23. Katz HR, Masket S, Lane SS, Sall K, Orr SC, Faulkner RD, et al. Absorption of topical moxifloxacin ophthalmic solution into human aqueous humor. Cornea. 2005 Nov;24(8):955–8.
24. Solomon R, Donnenfeld ED, Perry HD, Snyder RW, Nedrud C, Stein J, et al. Penetration of topically applied gatifloxacin 0.3%, moxifloxacin 0.5%, and ciprofloxacin 0.3% into the aqueous humor. Ophthalmology. 2005 Mar;112(3):466–9.
25. Güngör SG, Akova YA, Bozkurt A, Yasar Ü, Babaoğlu MÖ, Çetinkaya A, et al. Aqueous humour penetration of moxifloxocin and gatifloxacin eye drops in different dosing regimens before phacoemulsification surgery. Br J Ophthalmol. 2011 Sep;95(9):1272–5.
26. Kim DH, Stark WJ, O’Brien TP, Dick JD. Aqueous penetration and biological activity of moxifloxacin 0.5% ophthalmic solution and gatifloxacin 0.3% solution in cataract surgery patients. Ophthalmology. 2005 Nov;112(11):1992–6.
27. Ong-Tone L. Aqueous humor penetration of gatifloxacin and moxifloxacin eyedrops given by different methods before cataract surgery. J Cataract Refract Surg. 2007 Jan;33(1):59–62.
28. McCulley JP, Caudle D, Aronowicz JD, Shine WE. Fourth-generation fluoroquinolone penetration into the aqueous humor in humans. Ophthalmology. 2006 Jun;113(6):955–9.
29. Holland EJ, Lane SS, Kim T, Raizman M, Dunn S. Ocular penetration and pharmacokinetics of topical gatifloxacin 0.3% and moxifloxacin 0.5% ophthalmic solutions after keratoplasty. Cornea. 2008 Apr;27(3):314–9.
30. Hariprasad SM, Mieler WF, Shah GK, Blinder KJ, Apte RS, Holekamp NM, et al. HUMAN INTRAOCULAR PENETRATION PHARMACOKINETICS OF MOXIFLOXACIN 0.5% VIA TOPICAL AND COLLAGEN SHIELD ROUTES OF ADMINISTRATION. Trans Am Ophthalmol Soc. 2004 Dec;102:149–58.

31. Costello P, Bakri SJ, Beer PM, Singh RJ, Falk NS, Peters GB, et al. Vitreous penetration of topical moxifloxacin and gatifloxacin in humans. Retina Phila Pa. 2006 Feb;26(2):191–5.
32. Torkildsen G, Proksch JW, Shapiro A, Lynch SK, Comstock TL. Concentrations of besifloxacin, gatifloxacin, and moxifloxacin in human conjunctiva after topical ocular administration. Clin Ophthalmol Auckl NZ. 2010;4:331–41.
33. Wagner RS, Abelson MB, Shapiro A, Torkildsen G. Evaluation of moxifloxacin, ciprofloxacin, gatifloxacin, ofloxacin, and levofloxacin concentrations in human conjunctival tissue. Arch Ophthalmol Chic Ill 1960. 2005 Sep;123(9):1282–3.
34. Abul Kalam M, Sultana Y, Ali A, Aqil M, Mishra AK, Chuttani K, et al. Part II: Enhancement of transcorneal delivery of gatifloxacin by solid lipid nanoparticles in comparison to commercial aqueous eye drops. J Biomed Mater Res A. 2013 Jun;101(6):1828–36.
35. Kalam MA, Alshamsan A, Aljuffali IA, Mishra AK, Sultana Y. Delivery of gatifloxacin using microemulsion as vehicle: formulation, evaluation, transcorneal permeation and aqueous humor drug determination. Drug Deliv. 2016;23(3):896–907.
36. Liu Z, Yang X, Li X, Pan W, Li J. Study on the Ocular Pharmacokinetics of Ion-Activated In Situ Gelling Ophthalmic Delivery System for Gatifloxacin by Microdialysis. Drug Dev Ind Pharm. 2007;
37. Spadaro A, Pappalardo M. Preclinical Pharmacokinetic Evaluation of Gatifloxacin Mucoadhesive Formulations in Rabbit Eye using HPLC. J Pharm Sci. 2015;7:5.
38. Kobayakawa S, Ooki K, Tsuji A, Tochikubo T. Aqueous humor penetration of fourth-generation fluoroquinolone ophthalmic solutions given by multiple administration in a rabbit model. J Infect Chemother Off J Jpn Soc Chemother. 2009 Aug;15(4):209–13.
39. Liu X, Wang N, Wang Y, Ma C, Ma L, Gao L, et al. Determination of drug concentration in aqueous humor of cataract patients administered gatifloxacin ophthalmic gel. Chin Med J (Engl). 2010 Aug 5;123(15):2105–10.
40. Teshigawara T, Hata S, Hayashi T, Watanabe Y, Itoh Y, Hitoi K, et al. Penetration of gatifloxacin eye drops into the aqueous humor in humans. Ocul Immunol Inflamm. 2007 Aug;15(4):309–13.
41. Price MO, Quillin C, Price FW. Effect of gatifloxacin ophthalmic solution 0.3% on human corneal endothelial cell density and aqueous humor gatifloxacin concentration. Curr Eye Res. 2005 Jul;30(7):563–7.
