# Supplementary material for: Clinical Ocular Exposure Extrapolation for Ophthalmic Solutions Using PBPK Modeling and Simulation
Source: Pharm Res. 2022 Sep 23;40(2):431–47. doi: 10.1007/s11095-022-03390-z (PMC9944674; doi:10.1007/s11095-022-03390-z)
Supplement: Supplementary file 3 — Supplementary file3 (PDF 581 KB) [file 11095_2022_3390_MOESM3_ESM.pdf]

Clinical Ocular Exposure Extrapolation for Ophthalmic Solutions Using PBPK Modeling and Simulation

Maxime Le Merdy[1], Farah AlQaraghuli[1], Ming-Liang Tan[2], Ross Walenga[2], Andrew Babiskin[2], Liang Zhao[2], Viera Lukacova[1]

1: Simulations Plus, Inc., 42505 10th Street West, Lancaster, California 93534, USA.
2: Division of Quantitative Methods and Modeling, Office of Research and Standards, Office of Generic Drugs, Center for Drug Evaluation and Research, U.S. Food and Drug Administration, 10903 New Hampshire Avenue, Silver Spring, MD 20993, USA

Supplementary material 3: Preclinical OCAT Results

Figure 1: Observed (squares) and Simulated (lines) Iris Ciliary Body Concentration Time Course in DB Rabbit Following a Single Administration of Levofloxacin Solution (Study Lev.DB.1) 2
Figure 2: Observed (squares) and Simulated (line) Iris Ciliary Body Concentration Time Course in DB Rabbit Following a Single Administration of Moxifloxacin Solution (Study Mox.DB.1) 3
Figure 3: Observed (squares) and Simulated (line) Conjunctiva Concentration Time Course in DB Rabbit Following a Single Administration of Moxifloxacin Solution (Study Mox.DB.2)...................................... 3
Figure 4: Observed (squares) and Simulated (lines) Iris Ciliary Body Concentration Time Course in DB Rabbit Following Multiple Administration of Gatifloxacin Solution (Study Gat.DB.2) 4
Figure 5: Observed (squares) and Simulated (line) Conjunctiva Concentration Time Course in DB Rabbit Following a Single Administration of Gatifloxacin Solution (Study Gat.DB.1) 4
Figure 6: Observed (circles) and Simulated (lines) Aqueous Humor Concentration Time Course in Human Following Multiple Administration of Gatifloxacin Solution (Study Gat.Hum.11) 5

Levofloxacin

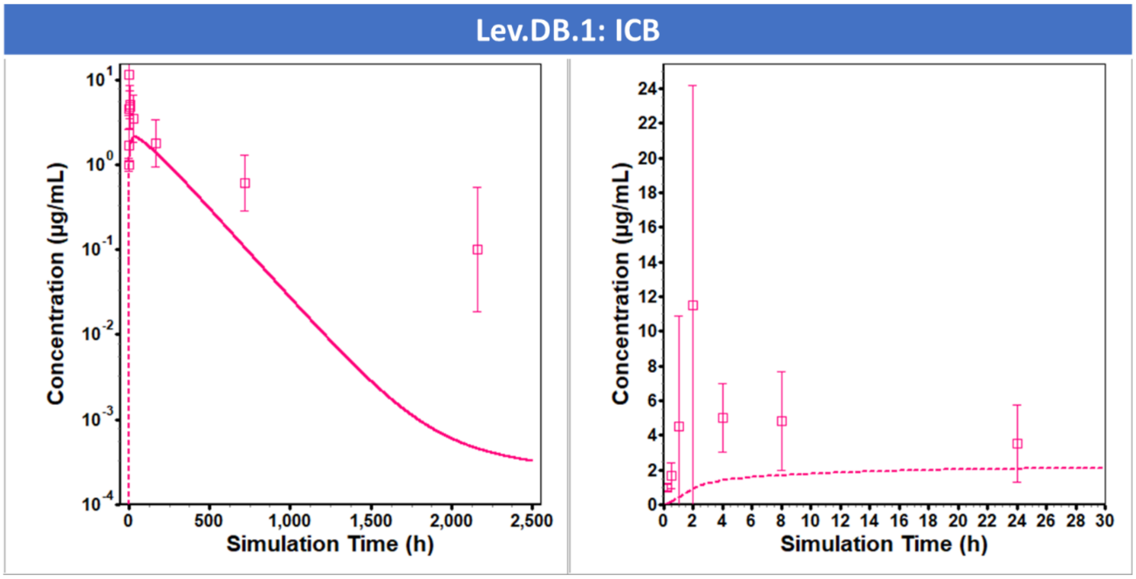

Figure 1: Observed (squares) and Simulated (lines) Iris Ciliary Body Concentration Time Course in DB Rabbit Following a Single Administration of Levofloxacin Solution (Study Lev.DB.1)

Moxifloxacin

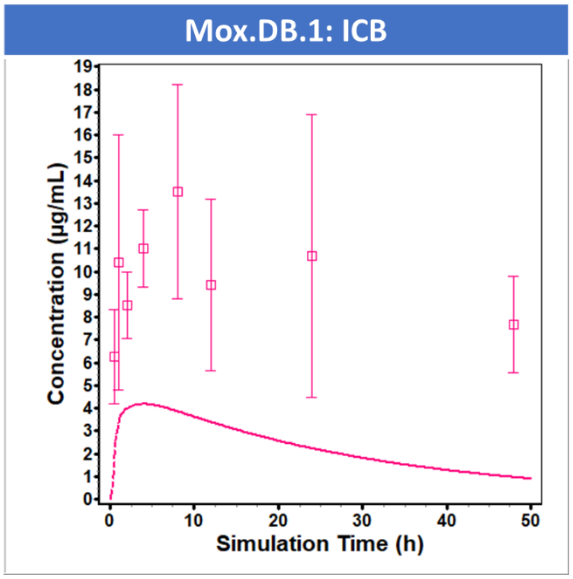

Figure 2: Observed (squares) and Simulated (line) Iris Ciliary Body Concentration Time Course in DB Rabbit Following a Single Administration of Moxifloxacin Solution (Study Mox.DB.1)

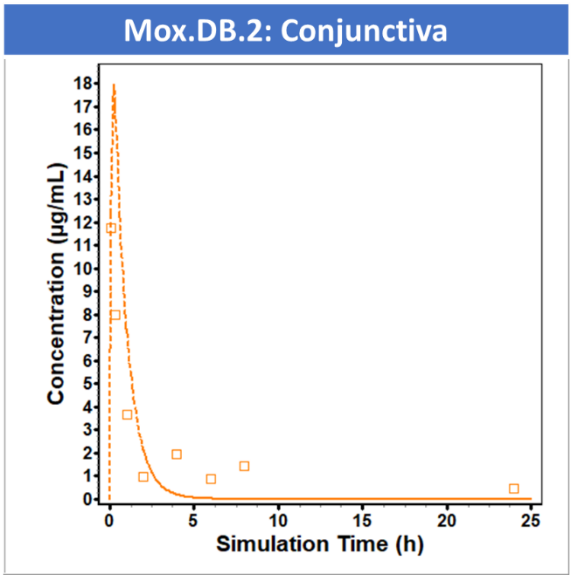

Figure 3: Observed (squares) and Simulated (line) Conjunctiva Concentration Time Course in DB Rabbit Following a Single Administration of Moxifloxacin Solution (Study Mox.DB.2)

Gatifloxacin

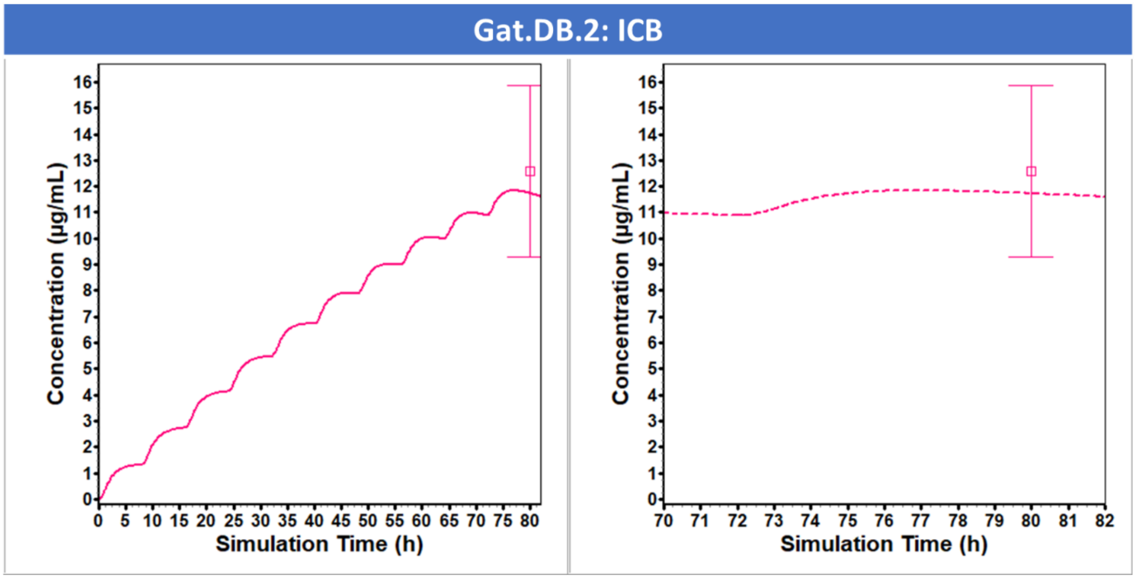

Figure 4: Observed (squares) and Simulated (lines) Iris Ciliary Body Concentration Time Course in DB Rabbit Following Multiple Administration of Gatifloxacin Solution (Study Gat.DB.2)

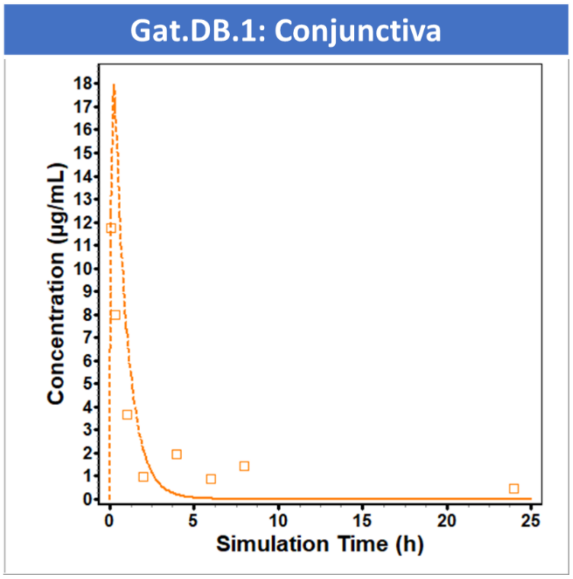

Figure 5: Observed (squares) and Simulated (line) Conjunctiva Concentration Time Course in DB Rabbit Following a Single Administration of Gatifloxacin Solution (Study Gat.DB.1)

Parameter Sensitivity Analysis

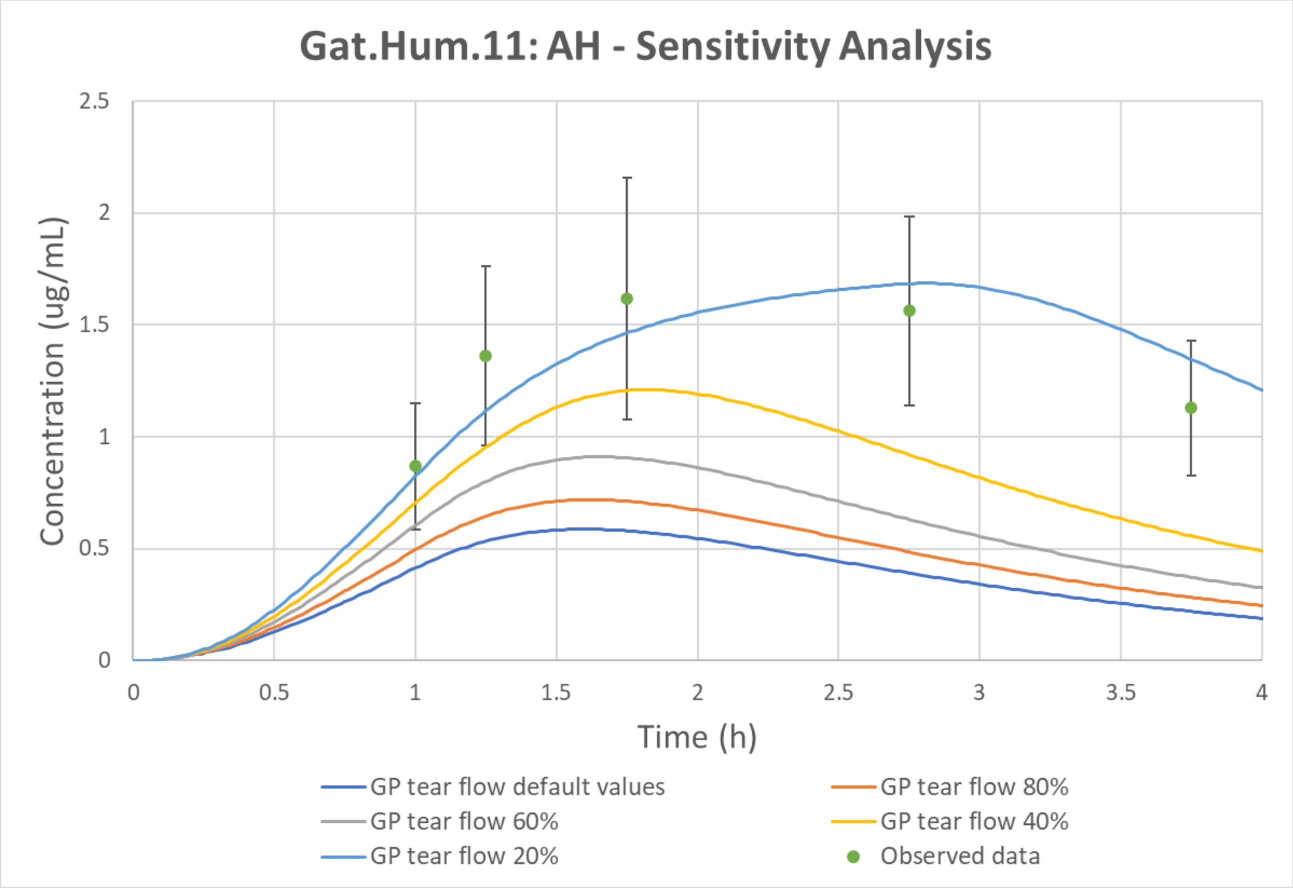

Figure 6: Observed (circles) and Simulated (lines) Aqueous Humor Concentration Time Course in Human Following Multiple Administration of Gatifloxacin Solution (Study Gat.Hum.11)
